# Systematic review and network meta-analysis of the effects of bioactive compounds on pain intensity and quality of life in neuropathic pain patients

**DOI:** 10.3389/fphar.2025.1656400

**Published:** 2025-10-17

**Authors:** Rong Tang, Chao-Yang Gong, Yu-Qian Liu, Yingbin Wang, Hai-Yu Zhou

**Affiliations:** ^1^ Department of Anesthesiology, The Second Hospital of Lanzhou University, Lanzhou, China; ^2^ Orthopaedics Key Laboratory of Gansu Province, Lanzhou, China; ^3^ Department of Orthopedics, The Second Hospital of Lanzhou University, Lanzhou, China

**Keywords:** bioactive compounds, pain intensity, quality of life, neuropathic pain patients, system review and meta-analysis

## Abstract

**Background:**

In recent years, a significant number of researchers have concentrated on bioactive compounds for the treatment of neuropathic pain. However, there remains a lack of compelling evidence to robustly support their therapeutic efficacy.

**Objective:**

This study aims to assess the impact of various bioactive compounds on pain intensity and quality of life in patients suffering from neuropathic pain by conducting a network meta-analysis.

**Methods:**

Researchers conducted a systematic search across five electronic databases—PubMed, EMBASE, Cochrane Library, the Cochrane Central Register of Controlled Trials, and Web of Science—from the inception of each database until April 2025. The methodological quality of the included studies was assessed using the Cochrane Risk of Bias assessment tool. Data analysis was subsequently performed using Stata MP 15.1 software. The primary outcome measures consisted of the following standardized assessment scales: Visual Analogue Scale (VAS), Neuropathic Pain Scale (NPS), Hospital Anxiety and Depression Scale (HADS), Patient Global Impression of Change (PGIC), and Leeds Sleep Evaluation Questionnaire (LSEQ). Treatment effects were ranked based on probability values derived from the Surface Under the Cumulative Ranking Curve (SUCRA).

**Results:**

Following the screening process, 20 eligible randomized controlled trials were included, involving a total of 2,471 patients and evaluating six distinct bioactive compound-based therapeutic interventions. The ranking of treatments, based on SUCRA values, indicated that tetrahydrocannabinol was associated with the highest likelihood of being the most effective option for reducing VAS scores (SUCRA: 88.2%). Furthermore, it consistently ranked favorably across other outcomes, including NPS (84.8%), PGIC (85.1%), and LSEQ (90.7%). Capsaicin was ranked as the most promising intervention for improving HADS scores (90.1%).

**Conclusion:**

This study offers valuable insights into the application of bioactive compounds for the management of neuropathic pain. However, the research also presents certain unavoidable limitations, such as heterogeneity among studies and the absence of direct comparative evidence for specific intervention measures. Future studies should include larger sample sizes, extended follow-up periods, and more rigorously designed randomized controlled trials to definitively establish the efficacy of bioactive compounds in patients with neuropathic pain patients.

**Systematic Review Registration:**

https://www.crd.york.ac.uk/PROSPERO/recorddashboard, identifier CRD420251041801.

## Introduction

Chronic pain, now recognized as a disease entity in its own right, represents a prevalent, costly, and debilitating clinical condition and is among the most frequent reasons for seeking healthcare (Gaskin and Richard, 2012; Pitcher et al., 2019; Steingrímsdóttir Ó et al., 2017). Neuropathic pain (NP), defined as pain resulting from damage or dysfunction of the somatosensory nervous system, affects 7%–10% of the population and is widely regarded as particularly challenging to manage (Colloca et al., 2017; van Hecke et al., 2014; Jensen et al., 2011). The complexity of NP necessitates a holistic and multidisciplinary approach that incorporates a range of therapeutic modalities, including pharmacotherapy, physical therapy, psychological interventions, and lifestyle modifications. The overarching objective of this comprehensive treatment strategy is to alleviate pain symptoms, improve quality of life, and optimize patient outcomes (Finnerup et al., 2015; Gilron et al., 2015). Based on the available evidence, pharmacotherapy constitutes a fundamental treatment modality for NP. Current clinical guidelines recommend tricyclic antidepressants, anticonvulsants (specifically pregabalin and gabapentin), and serotonin–norepinephrine reuptake inhibitors for use in NP pharmacotherapy. Opioids and local anesthetics are recommended as second-line therapeutic options (Finnerup et al., 2015; Moulin et al., 2007). However, treatment responses may vary considerably, and the occurrence of adverse effects frequently compromises patient adherence.

Bioactive compounds have garnered significant attention due to their favorable therapeutic effects, multi-target mechanisms, and minimal adverse effects. The compounds selected for inclusion in this study were primarily based on their well-documented biological activity in modulating NP pathways and reducing oxidative stress. Among these compounds, some are derived from plants, while others are commonly administered as synthetic or semi-synthetic supplements. Currently, the principal bioactive compounds recommended for the management of NP include cannabis, capsaicin, curcumin, and alpha-lipoic acid, which can be extracted from various natural plant sources such as cannabis sativa, capsicum annuum, turmeric, and spinach, among others (Berman et al., 2004; Agoons et al., 2020; Agathos et al., 2018; Asadi et al., 2019). Studies have shown that oral cannabinoids such as cannabis and tetrahydrocannabinol (THC) are effective in treating central (Svendsen et al., 2004; Rog et al., 2005) and peripheral NP (Nurmikko et al., 2007), rheumatoid arthritis (Blake et al., 2006) and fibromyalgia (Skrabek et al., 2008) when used alone or in combination. In a 12-week, randomized, double-blind, parallel-group clinical trial, the intention-to-treat population for efficacy analysis comprised 69 patients receiving clonidine and 70 patients receiving capsaicin. The results indicated that both drugs were highly effective in alleviating peripheral neuropathic pain at the 12-week follow-up. And no statistically significant difference in therapeutic efficacy was observed between the two treatment modalities (Kiani et al., 2015). Curcumin, the key bioactive compound found in turmeric, exhibits substantial potential for scavenging free radicals and mitigating oxidative stress due to its antioxidant properties. Research has demonstrated that compared with the placebo group, supplementation with curcumin over a 2-month period can significantly alleviate and reduce the severity of diabetic sensorimotor polyneuropathy in individuals with type 2 diabetes mellitus (Asadi et al., 2019). Alpha-lipoic acid, an essential co-enzyme for energy production in mitochondria, demonstrates substantial antioxidant properties and an effect on whole-body physiology (Evans and Goldfine, 2000), which is used as a dietary supplement and a pharmaceutical agent (Papanas and Ziegler, 2014). Alpha-lipoic acid exhibits superior absorption characteristics and is capable of diffusing efficiently in both extracellular and intracellular environments. Furthermore, it possesses the unique capacity to penetrate the blood-brain barrier, ensuring its accessibility to crucial neural tissues. It has been utilized in various models of oxidative stress, such as diabetes, ischemia-reperfusion injury, cataract formation, and neurodegenerative disorders (Packer et al., 1995).

Currently, there is a lack of evidence-based guidelines on which bioactive compounds are most effective in improving pain intensity and quality of life in patients with NP. Network meta-analysis (NMA) is a data-driven method that evaluates the effectiveness of diverse interventions through direct or indirect comparisons, thereby offering a ranked assessment of their relative effectiveness (Rouse et al., 2017). Accordingly, this study represents the first comprehensive NMA of randomized controlled trials (RCTs) on the treatment of NP with bioactive compounds, aiming to provide high-quality evidence to support clinical decision-making.

## Methods

The conductions of meta-analysis and systematic review rigorously followed Preferred Reporting Items for Systematic Reviews and Meta-Analyses (PRISMA) guidelines and was registered on PROSPERO (registration number: CRD420251041801). https://www.crd.york.ac.uk/PROSPERO/recorddashboard. Minor deviations from the PROSPERO protocol occurred during the conduct of this review. These deviations are summarized in Supplementary Table S1.

### Search strategy

Two of the authors (TR and GCY) conducted a comprehensive and systematic search of relevant studies from various electronic databases, encompassing PubMed, EMBASE, Cochrane Library, Cochrane Central Register of Controlled Trials, and Web of Science. This extensive search covered the period from the inception of each database until April 2025, ensuring a thorough examination of the available literature. The search strategy was developed based on intervention measures and patient types. The detailed search strategies are presented in Supplementary Table S2 (using PubMed as an example).

### Inclusion and exclusion criteria

Inclusion and exclusion criteria were established based on the PICOS framework: (P) Population: NP patients; (I) Intervention: bioactive compounds, which refers to exogenously administered, naturally occurring or semi-synthetic molecules with defined pharmacological activity. These compounds are used for the treatment of neuropathic pain. To enhance clarity, we categorized them into the following three subclasses based on their chemical nature and source: cannabinoids; vitamin-related compounds and botanical extracts and isolates. (C) Comparison: control groups receiving either usual treatment or placebo; (O) Outcomes: improvement in pain intensity and quality of life in NP patients, assessed using at least one of the following scales: VAS, NPS, LSEQ, HADS, and PGIC. (S) Study Type: RCTs.

In our study, we strictly predefined the primary outcome as changes in pain intensity based on the VAS or NPS scales, explicitly excluding those based on composite or proxy endpoints, to ensure outcome homogeneity and clarity of conclusions. Furthermore, we excluded studies characterized by incomplete or unrecorded data, as well as those involving non-RCTs, preclinical studies, animal experiments, systematic reviews, case reports, meta-analyses, conference abstracts, case analyses, review articles, letters, and the full text cannot be obtained.

### Study selection and methodological assessment

Two members of the research team (TR and LYQ) independently performed the literature selection process. Initially, the retrieved studies were imported into the reference management software Endnote X8, and duplicate entries were removed. Subsequently, the titles and abstracts were screened in accordance with the predefined inclusion criteria to exclude studies that clearly did not meet the requirements. Finally, the full texts of the remaining articles were obtained and critically reviewed to determine final inclusion. Throughout this process, any discrepancies in judgment were resolved through thorough discussion; when necessary, a third investigator (GCY) was consulted to facilitate consensus.

Two researchers (GCY and LYQ) independently evaluated the risk of bias in the included RCTs using the Cochrane Risk of Bias (ROB) assessment tool. We conducted a systematic evaluation of seven domains: (1) the random sequence generation, (2) allocation concealment, (3) performance bias (blinding of both researchers and participants), (4) Detection bias (blinding of outcome assessment), (5) the management of missing data, (6) reporting bias (selective reporting), and (7) other sources of bias (Higgins et al., 2011). The results extracted from each RCT were used until the consensus was reached between the two researchers. We resolved disagreements by discussing or negotiating with the third researcher (TR) until the consensus was reached.

### Data extraction

A self-designed standardized data extraction table was adopted to independently extract data. The extracted information includes the main author, country, year of publication, study type, number of patients, mean age of patients, details of the interventions, outcome measures assessing the effects of bioactive compounds on NP patients (VAS, NPS, LSEQ, HADS, PGIC). The inconsistencies in the data extraction process were resolved by rechecking the original data and consulting the third researcher (GCY).

The data were initially sourced from the tables as the primary extraction point. In instances where the data were incomplete, we reached out to the original author to acquire more information. In addition, we estimated the standard deviation (SD) using the formulas SD = Range/4 and SD = interquartile Range (IQR)/1.35 to include trials that only reported range and IQR, as detailed in the Cochrane Handbook for Systematic Reviews (Cumpston et al., 2019). Data reported with 95% confidence interval (CI) were also used to determine the range and convert to SD. If no average value was provided, the median was utilized to approximate its value (Hozo et al., 2005). When there is necessary data in the figures but the original data cannot be obtained from the author, we use the ImageJ software to extract the required data.

### Outcomes assessed

We employed the VAS and NPS as primary measures of pain intensity. Quality of life was assessed using the LSEQ, HADS, and PGIC. It is important to note that for the VAS, NPS, HADS, and PGIC, lower scores indicate better outcomes, whereas for the LSEQ, higher scores indicate better outcomes (Rowbotham et al., 1998). The secondary outcome was the incidence of adverse effects. Prior to analysis, we predefined minimal clinically important differences (MCIDs) for interpreting outcome changes: a reduction of 2 points was considered clinically meaningful for VAS, NPS, and HADS, while a 1-point change was used for PGIC and LSEQ, based on established thresholds in chronic pain populations (Dworkin et al., 2008).

### Statistical methods

Continuous outcomes were expressed as mean differences (MD) or standardized mean differences (SMD) with 95% CI, depending on the uniformity of measurement scales across studies. Dichotomous outcomes were analyzed using risk ratios (RR) with 95% CI. The direction of effects was standardized such that for VAS, NPS, HADS, and PGIC, lower scores indicated improvement, while for LSEQ, higher scores denoted benefit.

Network meta-analysis was performed using Stata MP 15.1, in accordance with PRISMA guidelines (Moher et al., 2015). Network diagrams were generated where node size and edge thickness corresponded to the number of studies and direct comparison respectively (Chaimani et al., 2013). Results are summarized in league tables, and treatments were ranked using SUCRA values, interpreted with consideration of evidence quality and clinical relevance.

Publication bias was assessed via visual inspection of network funnel plots and Egger’s test for small-study effects, with p < 0.05 indicating potential asymmetry.

### Assessment of heterogeneity and inconsistency

Heterogeneity among studies was assessed using the I^2^ statistic. An I^2^ value > 50% alongside a p-value <0.05 indicated substantial heterogeneity, warranting the use of a random-effects model for data synthesis. Otherwise, a fixed-effect model was applied. Given the anticipated clinical and methodological diversity across studies, a random-effects model was prioritized as the primary analytical framework. This approach more conservatively accounts for variability in true effect sizes and yields more robust conclusions.

To explore potential sources of heterogeneity, pre-specified subgroup analyses were conducted based on route of administration, pain etiology, and intervention duration, as these factors are clinically relevant to treatment response.

Inconsistency in the network was evaluated globally using the design-by-treatment interaction model and locally using node-splitting analysis to identify discrepancies between direct and indirect evidence.

### Sensitivity analysis

To evaluate the robustness of the primary results, sensitivity analyses were conducted by sequentially excluding studies with a high risk of bias, those with imputed or digitally extracted data, or those using vague or non-standard unit scales. The consistency of effect estimates, confidence intervals, and statistical significance with the main findings was evaluated.

Additionally, to address clinical heterogeneity in the administration of vitamin D (oral vs. intramuscular), the node was split into two distinct entities. The network meta-analysis was rerun using the same model and priors. Global and local inconsistency measures were examined, and results were compared to those of the primary analysis to assess the stability of the conclusions.

### GRADE grading

We employed the Grade of Recommendations, Assessment, Development, and Evaluation (GRADE) guidelines to appraise the strength of the evidence gleaned from the incorporated trials. The GRADE methodology meticulously elucidates the various elements impinging on the quality of evidence and provides quantitative criteria for grading (Guyatt et al., 2011; Mendoza et al., 2017). Evidence from randomized trials starts as high certainty but can be downgraded for five reasons: risk of bias, inconsistency, indirectness, imprecision, and publication bias. The GRADE tool classifies the strength of accumulated evidence into four levels: high, moderate, low, or very low quality.

## Results

**FIGURE 1:**
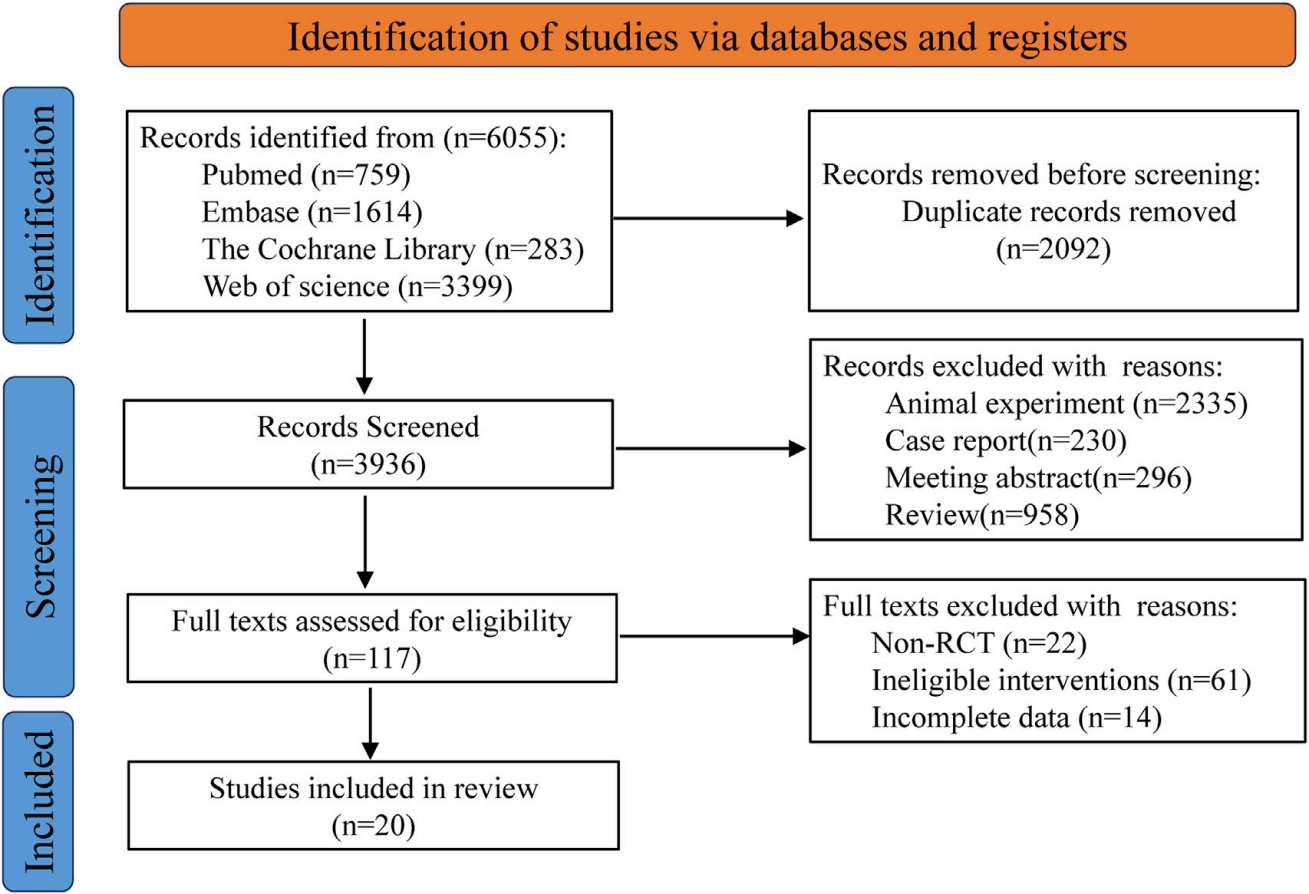
Flow diagram of literature selection.

Based on the predefined search strategy, a total of 6,055 records were identified from multiple electronic databases. Following the use of reference management software to remove 2,092 duplicate entries, the titles and abstracts of the remaining 3,936 records were carefully reviewed. This preliminary screening process led to the exclusion of 3,819 records that did not meet the inclusion criteria. The full texts of the remaining 117 articles were then thoroughly evaluated, resulting in the exclusion of an additional 97 articles due to reasons such as non- RCTs, incomplete data reporting, or interventions outside the scope of the study. Ultimately, 20 articles were determined to meet all eligibility criteria and were included in the final analysis (Berman et al., 2004; Agoons et al., 2020; Agathos et al., 2018; Asadi et al., 2019; Rog et al., 2005; Kiani et al., 2015; Abbas et al., 2022; Cruccu et al., 2018; Mankowski et al., 2017; Mehta et al., 2021; Nadro et al., 2021; Papaioannou, 2018; Sa et al., 2020; Wallace et al., 2015; Ware et al., 2010; Weizman et al., 2018; Wilsey et al., 2013; Wilsey et al., 2008; Wilsey et al., 2016; Ziegler et al., 1999). The flowchart for retrieving and filtering records is shown in Figure 1, and the reasons for exclusion are summarized. No other studies that met the conditions were found after manual supplementary search.

### Quality assessment of the included studies

**FIGURE 2:**
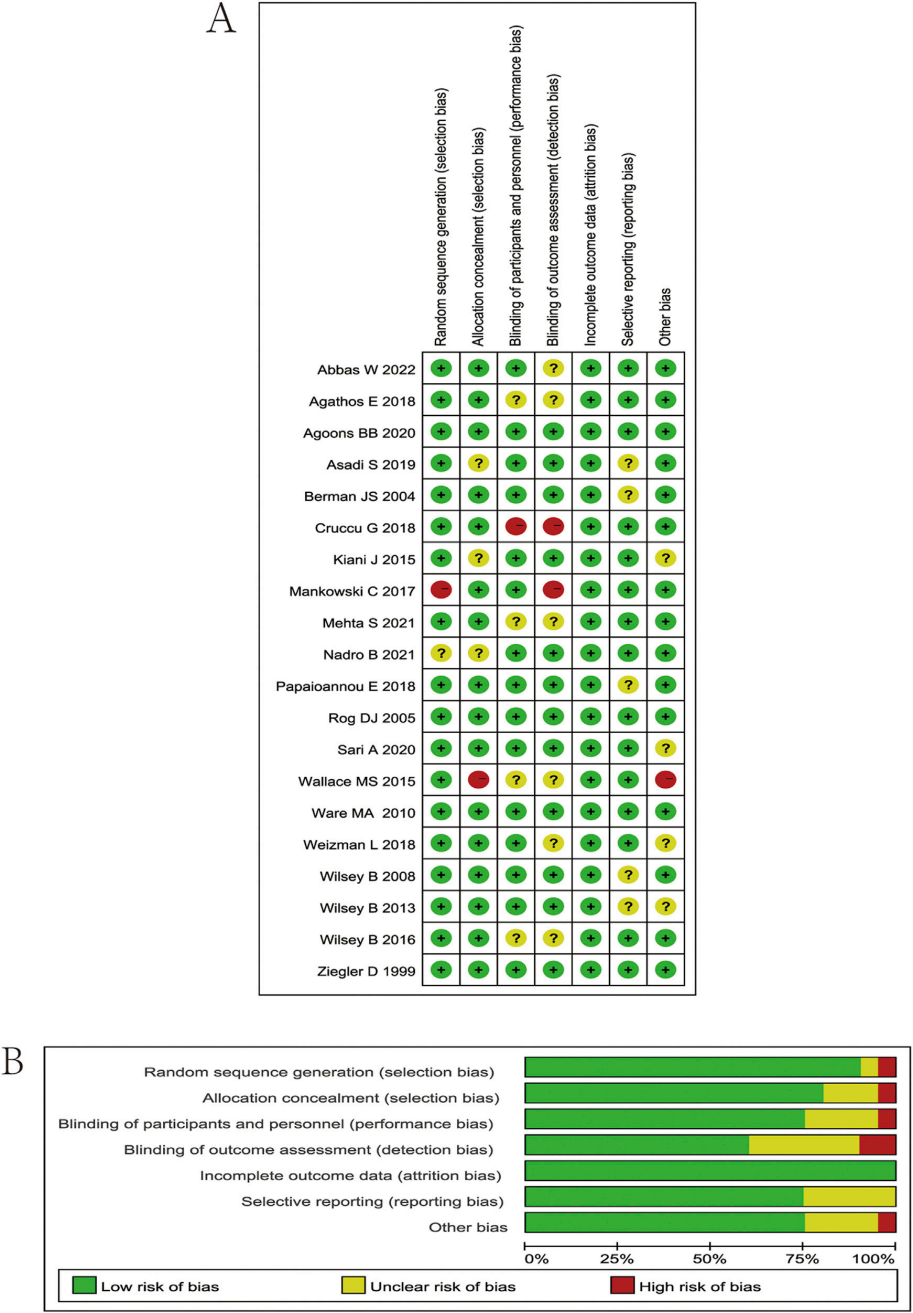
**(A)** Risk of bias summary. Review authors’ judgments about each risk of bias item for each included study. Green circle, low risk of bias; red circle, high risk of bias; yellow circle, unclear risk of bias. **(B)** Risk of bias graph. Which presents the percentage of studies rated as low, high, or having some concerns for each bias domain across all included studies.

The researchers’ quality assessment of the included trials is shown in Figure 2. Random allocation procedures were explicitly reported in some trials, whereas others lacked sufficient detail to adequately assess the risk of bias. In cases where the concealment of the randomization process and the allocation sequence could not be verified, a conservative approach was adopted in evaluating the risk of bias. All 20 included studies were randomized controlled trials (RCTs), with 9 classified as low risk, 8 as moderate risk, and 3 as high risk, suggesting that the overall methodological quality of the included studies was acceptable.

### Characteristics of the included studies

Our analysis included 20 RCTs, encompassing 2471 patients. Interventions in the treatment group included cannabis, THC, capsaicin, curcumin, alpha-lipoic acid, and vitamin D. 14 studies utilized VAS as an outcome indicator, 11 studies reported NPS, 7 studies reported HADS, 11 studies reported PGIC, and 3 studies reported LSEQ. The characteristics of the included studies are shown in Table 1.

**TABLE 1:** Characteristics of the studies included in the meta-analysis. Abbreviations: N, number; E, experimental group; C, control group; PNP, peripheral neuropathic pain; CNP, central neuropathic pain; DNP, diabetic neuropathic pain; THC, tetrahydrocannabinol; ① Visual Analogue Scale (VAS); ② Neuropathic Pain Scale (NPS); ③ Hospital Anxiety and Depression Scale (HADS); ④ Patient Global Impression of Change (PGIC); ⑤ Leeds Sleep Evaluation Questionnaire (LSEQ).

Author	year	Pain cause	N	Age	Experimental interventions	Control interventions	Intervention time	Outcome	The route of administration	Exact dosages
Ware MA	2010	PNP	E: 21	E: 45.4 ± 12.3	Cannabis	Placebo	8 weeks	①③⑤	Inhaled	75 mg/day
			C: 22	C: 45.4 ± 12.3						
Weizman L	2018	PNP	E: 15	E: 33.3 ± 3.9	THC	Placebo	6 weeks	①②	Inhaled	15.4 ± 2.2 mg/day
			C: 15	C: 33.3 ± 3.9						
Rog DJ	2005	CNP	E: 32	E: 49.2 ± 8.3	Cannabis	Placebo	5 weeks	①②③④⑤	Inhaled	67.5 ± 16.2 mg/day
			C: 34	C: 48.1 ± 9.7						
Berman JS	2004	CNP	E: 48	E: 39 (23–63)	THC	Placebo	8 weeks	①②⑤	Inhaled	54 ± 13.5 mg/day
			C: 47	C: 39 (23–63)						
Wilsey B	2013	CNP	E: 38	E: 50 ± 11	THC	Placebo	4 weeks	①②④	Inhaled	Not specified
			C: 38	C: 50 ± 11						
Wilsey B	2016	CNP	E: 40	E: 46.4 ± 13.6	THC	Placebo	4 weeks	①②④	Inhaled	Not specified
			C: 40	C: 46.4 ± 13.6						
Wilsey B	2008	CNP	E: 34	E: 46(21–71)	cannabis	Placebo	4 weeks	①③④	Inhaled	Not specified
			C: 33	C: 46(21–71)						
Wallace MS	2015	DNP	E: 16	E: 56.9 ± 8.2	THC	Placebo	6 weeks	①③	Inhaled	4–28 mg/day
			C: 16	E: 55.9 ± 8.7						
Cruccu G	2018	PNP	E: 253	E: 54.5 ± 11.7	capsaicin	Usual treatment	8 weeks	①	Topical	8% patch(1-4patches a day)
			C: 235	C: 55.5 ± 10.6						
Kiani J	2015	DPN	E: 39	E: 56.49 ± 10.25	capsaicin	Usual treatment	12 weeks	①	Topical	0.75% cream (3 times a day)
			C: 54	C: 56.88 ± 9.54						
Mankowski C	2017	PNP	E: 290	E: 61 (21–75)	capsaicin	Usual treatment	8 weeks	①④	Topical	8% patch (1-4patches a day)
			C: 130	C: 63 (22–73)						
Agoons BB	2020	DPN	E: 11	E: 56.0 (50–60)	capsaicin	Placebo	8 weeks	①③④	Topical	0.75% gel (3 times a day)
			C: 11	C: 58.0 (50–62)						
Papaioannou E	2018	DPN	E: 37	E: 53.5 ± 11.3	capsaicine	Usual treatment	8 weeks	①④	Topical	8% patch (1-4patches a day)
			C: 37	C: 54.2 ± 10.7						
Nadro B	2021	DPN	E: 32	E: 64.15 ± 8.66	alpha-lipoic acid	Usual treatment	6weeks	②	Oral	600 mg/day
			C: 22	C: 63.58 ± 5.12						
Agathos E	2018	DPN	E: 72	E: 65.2 ± 8.4	alpha-lipoic acid	Placebo	24 weeks	②	Oral	600 mg/day
			C: 72	C: 65.2 ± 8.4						
Ziegler D	1999	DPN	E: 165	E: 56.5 ± 7.1	alpha-lipoic acid	Placebo	24 weeks	①④	Oral	600 mg/day
			C: 165	C: 57.3 ± 5.5						
Mehta S	2021	DPN	E: 50	E: 50 ± 6	vitamin D	Usual treatment	24 weeks	②④	Oral	60,000 IU/week
			C: 50	C: 50 ± 6						
Sari A	2020	DPN	E: 32	E: 63.49 ± 9.79	vitamin D	Placebo	12 weeks	②③④	Intramuscularly	300,000 IU
			C: 25	C: 62.16 ± 9.10						
Asadi S	2019	DPN	E: 40	53.3 ± 6.5	curcumin	Placebo	8 weeks	②④	Oral	80 mg/day
			C: 40	54.6 ± 6.2						
Abbas W	2022	DPN	E: 35	E: 48.4 ± 12.6	curcumin	Usual treatment	8 weeks	②③	Oral	80 mg/day
			C: 37	C: 49.3 ± 12.9						

### Model specification

We calculated the global heterogeneity variance and I^2^ statistics. The results show that there is moderate to high heterogeneity for the outcomes of VAS, NPS and PGIC, which supports the necessity of using the random effects models. For HADS and LSEQ, since the I^2^<50%, the fixed-effects models were employed. In response to test the robustness of the results, we also conducted the sensitivity analysis. The results show that the ranking and statistical conclusions of the effects under the two models remain consistent, which enhances the reliability of our main conclusion. Further details can be found in Supplementary Table S3.

### Evidence network structure and contribution

**FIGURE 3:**
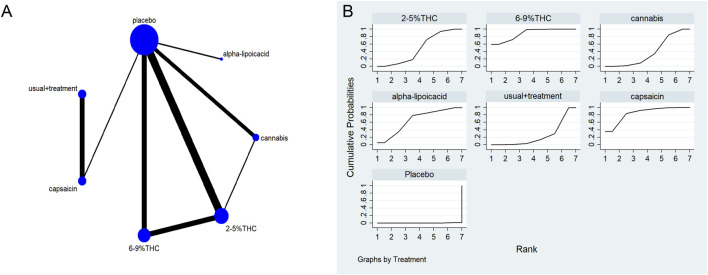
**(A)** Network diagram for VAS outcome. The network plot illustrates the available direct comparisons between interventions. Each node represents an intervention, with its size proportional to the number of studies investigating that intervention. The connecting lines between nodes represent direct head-to-head comparisons, and the thickness of each line is proportional to the number of trials for that specific comparison. **(B)** Surface Under the Cumulative Ranking Curve (SUCRA) plot for VAS. Treatments are ranked along the X-axis from best (left) to worst (right) based on their mean efficacy. The Y-axis represents the cumulative probability of a treatment being ranked at each position. The SUCRA for each treatment is presented as a numerical value (higher SUCRA values indicate a more effective treatment).

**FIGURE 4:**
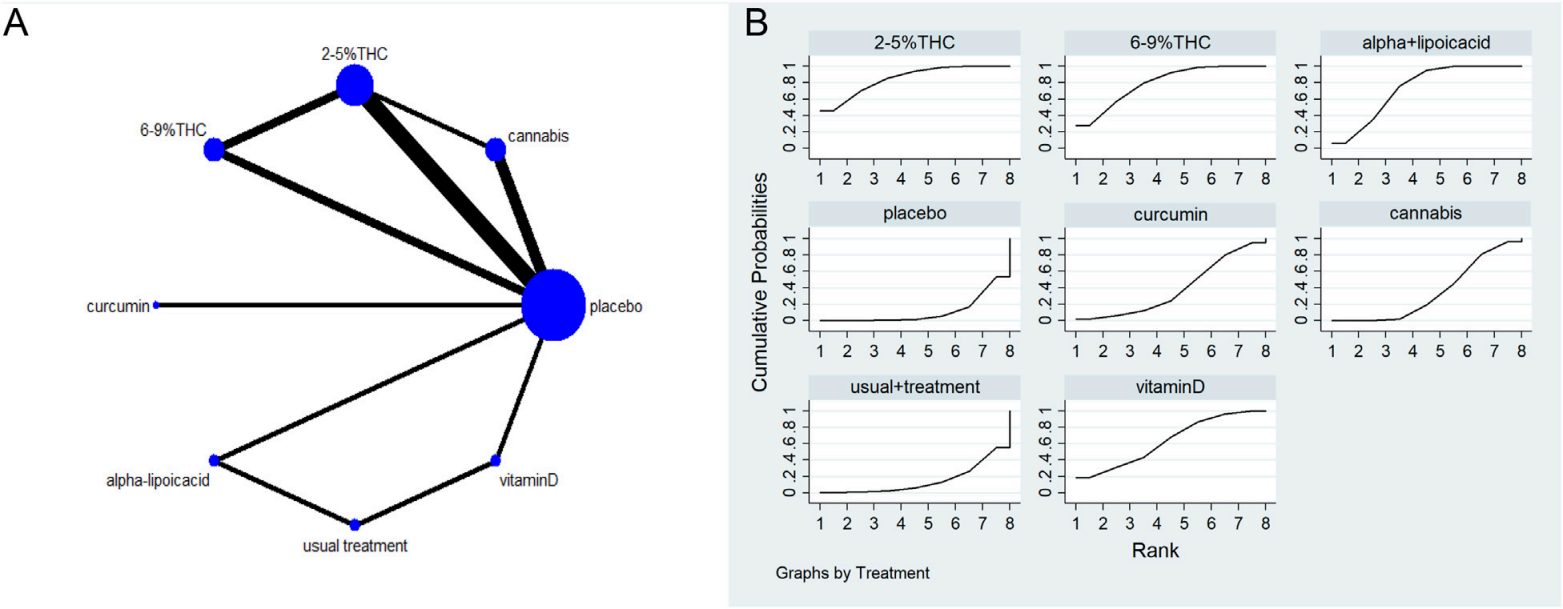
**(A)** Network diagram for NPS Outcome. The network plot illustrates the available direct comparisons between interventions. Each node represents an intervention, with its size proportional to the number of studies investigating that intervention. The connecting lines between nodes represent direct head-to-head comparisons, and the thickness of each line is proportional to the number of trials for that specific comparison. **(B)** Surface Under the Cumulative Ranking Curve (SUCRA) plot for NPS. Treatments are ranked along the X-axis from best (left) to worst (right) based on their mean efficacy. The Y-axis represents the cumulative probability of a treatment being ranked at each position. The SUCRA for each treatment is presented as a numerical value (higher SUCRA values indicate a more effective treatment).

**FIGURE 5:**
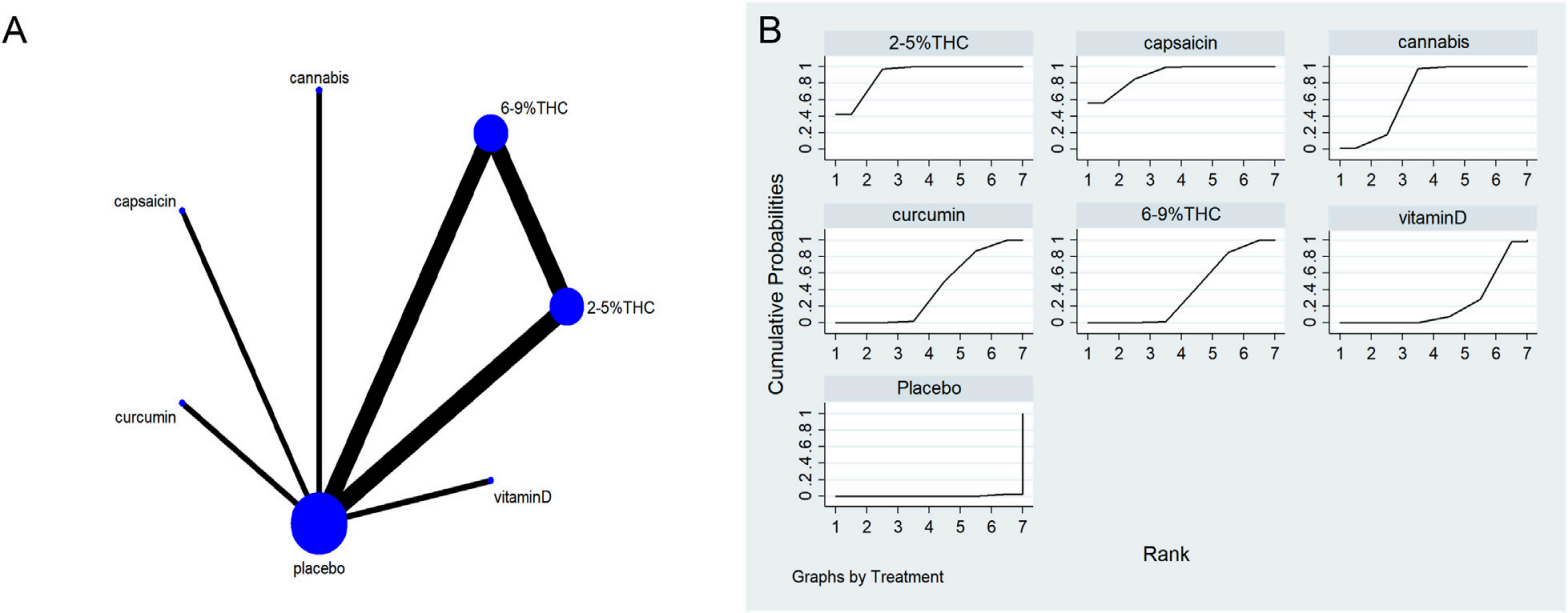
**(A)** Network diagram for HADS Outcome. The network plot illustrates the available direct comparisons between interventions. Each node represents an intervention, with its size proportional to the number of studies investigating that intervention. The connecting lines between nodes represent direct head-to-head comparisons, and the thickness of each line is proportional to the number of trials for that specific comparison. **(B)** Surface Under the Cumulative Ranking Curve (SUCRA) plot for HADS. Treatments are ranked along the X-axis from best (left) to worst (right) based on their mean efficacy. The Y-axis represents the cumulative probability of a treatment being ranked at each position. The SUCRA for each treatment is presented as a numerical value (higher SUCRA values indicate a more effective treatment).

**FIGURE 6:**
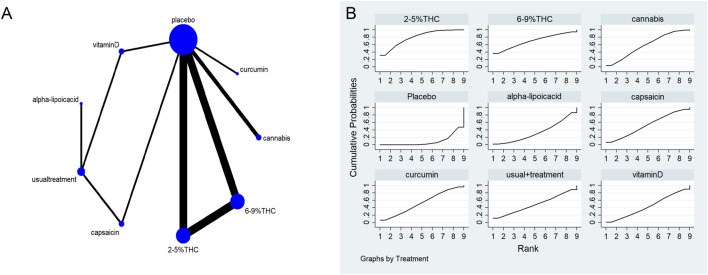
**(A)** Network diagram for PGIC Outcome. The network plot illustrates the available direct comparisons between interventions. Each node represents an intervention, with its size proportional to the number of studies investigating that intervention. The connecting lines between nodes represent direct head-to-head comparisons, and the thickness of each line is proportional to the number of trials for that specific comparison. **(B)** Surface Under the Cumulative Ranking Curve (SUCRA) plot for PGIC. Treatments are ranked along the X-axis from best (left) to worst (right) based on their mean efficacy. The Y-axis represents the cumulative probability of a treatment being ranked at each position. The SUCRA for each treatment is presented as a numerical value (higher SUCRA values indicate a more effective treatment).

**FIGURE 7:**
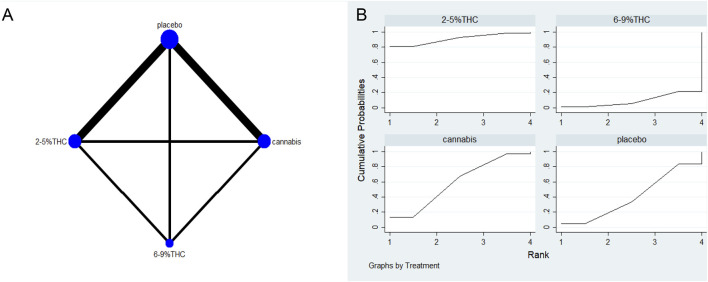
**(A)** Network diagram for LSEQ Outcome. The network plot illustrates the available direct comparisons between interventions. Each node represents an intervention, with its size proportional to the number of studies investigating that intervention. The connecting lines between nodes represent direct head-to-head comparisons, and the thickness of each line is proportional to the number of trials for that specific comparison. **(B)** Surface Under the Cumulative Ranking Curve (SUCRA) plot for LSEQ. Treatments are ranked along the X-axis from best (left) to worst (right) based on their mean efficacy. The Y-axis represents the cumulative probability of a treatment being ranked at each position. The SUCRA for each treatment is presented as a numerical value (higher SUCRA values indicate a more effective treatment).

The full network meta-evidence diagrams will be shown in Figures 3A, 4A, 5A, 6A, 7A. These diagrams visually present the structure of the evidence network, including the direct comparison relationships of all treatments. In addition, the contribution plots will be presented in Supplementary Figures S1–S5. Which details the proportion of information contribution of each direct comparison to the overall estimated value of the network. This helps to identify the most influential direct evidence and the links with sparse evidence.

### The visual analog scale (VAS)

The network meta-evidence diagram of VAS is shown in Figure 3A. The consistency of the P-values of all comparative analyses was systematically evaluated. The result showed that all *p*-values from the direct and indirect comparisons between studies were greater than 0.05, this indicates a satisfactory consistency among the studies. Additional details can be found in Supplementary Table S4 **.**

The results of the NMA indicated that 6%–9% THC (SMD = −1.74, 95% CI: -3.01 to −0.46), capsaicin (SMD = −1.45, 95% CI: -3.40 to −0.50), alpha-lipoic acid (SMD = −1.07, 95% CI: -3.12 to −0.99) were more effective in reducing VAS scores compared to the control group. The probability rankings for different bioactive compounds in reducing VAS scores showed that 6%–9% THC had the highest SUCRA value (88.2%, as shown in Figure 3B), ranking first among all interventions. A comparative analysis of different interventions is presented in Table 2.

**TABLE 2:** Treatments ranked by SUCRA values and the league table of their relative effectiveness in terms of VAS. Treatments are ordered by SUCRA values (higher percentage indicates greater probability of being effective). The league table presents pairwise comparisons as standardized mean differences with 95% confidence interval, Results that do not overlap with zero are considered statistically significant. Lower VAS scores signify better outcomes. Negative values indicate that column therapy is superior to row therapy. The results derived from the random-effects models.

**6–9%THC (SUCRA 88.2%)**						
−0.28 (−2.51, 1.94)	**Capsaicin (SUCRA 84.5%)**					
−0.67 (−2.99, 1.64)	−0.39 (−1.04, 0.26)	**Alpha-lipoic acid (SUCRA 65.7%)**				
**−1.56 (-2.87, -0.25)**	−1.27 (−3.25,0.70)	−0.89 (−2.96, 1.19)	**2–5%THC (SUCRA 48.6%)**			
**−2.09 (-3.47, -0.71)**	−1.80 (−3.83, 0.22)	−1.42 (−3.54, 0.71)	0.53 (−0.37, 1.43)	**Cannabis (SUCRA 38.3%)**		
**−3.08 (-4.25, -1.91)**	−2.80 (−4.69, 0.91)	−2.41 (−4.40, 0.41)	−1.52 (−2.10, 0.94)	0.99 (0.27, 1.72)	**Usual treatment (SUCRA 24.4%)**	
**−1.74 (-3.01, -0.46)**	**−1.45 (-3.40, -0.50)**	**−1.07 (-3.12, -0.99)**	0.18 (−0.40, 0.76)	0.35 (−0.47, 1.18)	1.34 (0.85, 1.84)	**Placebo (SUCRA 0.2%)**

Bold font represents statistical difference.

### The neuropathic pain scale (NPS)

The network meta-evidence diagram for NPS is shown in Figure 4A. All *p*-values for the indirect and direct comparisons between studies were exceeded 0.05, indicating that the consistency of effects among the studies is acceptable. Further details can be found in Supplementary Table S5.

The NMA results indicate that in improving NPS scores, 2–5%THC (MD = −1.83, 95% CI: -3.34 to −0.33), 6–9%THC (MD = −1.59, 95% CI: -3.13 to −0.06), and alpha-lipoic acid (MD = −1.36, 95% CI: -2.47 to −0.24) were superior to the placebo group. Additionally, regarding probability rankings among different bioactive compounds for improving NPS scores, 2–5%THC ranked first in SUCRA (SUCRA = 84.8%, as shown in Figure 4B). A comparison of the different interventions is provided in Table 3.

**TABLE 3:** Treatments ranked by SUCRA values and the league table of their relative effectiveness in terms of NPS. Treatments are ordered by SUCRA values (higher percentage indicates greater probability of being effective). The league table presents pairwise comparisons as mean differences with 95% confidence interval, Results that do not overlap with zero are considered statistically significant. Lower NPS scores signify better outcomes. Negative values indicate that column therapy is superior to row therapy. The results derived from the random-effects models.

**2–5%THC (SUCRA 84.8%)**							
−0.24 (−2.15,1.67)	**6–9%THC (SUCRA 79.3%)**						
−0.48 (−1.99,1.03)	−0.24 (−1.51,1.03)	**Alpha-lipoic acid (SUCRA 73.1%)**					
−0.85 (−3.60,1.89)	−0.62 (−3.24,2.01)	−0.38 (−2.67,1.92)	**VitaminD (SUCRA 63.3%)**				
−1.74 (−4.25,0.76)	−1.50 (−3.87,0.87)	−1.26 (−3.27,0.74)	−0.89 (−2.88,1.10)	**Curcumin (SUCRA 38.7%)**			
**−3.07 (-5.56, -0.58)**	**−2.83 (-5.18, -0.48)**	−2.59 (−4.57,0.61)	−2.22 (−4.21,0.22)	−1.33 (−3.63,0.97)	**Cannabis (SUCRA 34.8%)**		
**−3.04 (-5.81, -0.27)**	**−2.80 (-5.45, -0.15)**	**−2.56 (-4.88, -0.24)**	−2.18 (−5.45,1.08)	−1.30 (−4.36,1.77)	0.03 (−3.02,3.08)	**Usual treatment (SUCRA 14.7%)**	
**−1.83 (-3.34, -0.33)**	**−1.59 (-3.13, -0.06)**	**−1.36 (-2.47, -0.24)**	−0.98 (−3.53,1.57)	−0.09 (−2.38,2.20)	1.24 (−1.03,3.50)	1.20 (−1.37,3.78)	**Placebo (SUCRA 11.2%)**

Bold font represents statistical difference.

### Hospital anxiety and depression scale (HADS)

The network meta-evidence diagram is shown in Figure 5A. Consistency tests for direct and indirect comparisons among all studies showed that the p-value were greater than 0.05; further details are provided in Supplementary Table S6.

The NMA results showed that all bioactive compounds treatments were superior to placebo in improving HADS scores, details are provided in Table 4. Furthermore, in terms of probability rankings among different bioactive compounds for improving HADS scores, capsaicin ranked first in SUCRA (SUCRA = 90.1%, as shown in Figure 5B).

**TABLE 4:** Treatments ranked by SUCRA values and the league table of their relative effectiveness in terms of HADS. Treatments are ordered by SUCRA values (higher percentage indicates greater probability of being effective). The league table presents pairwise comparisons as mean differences with 95% confidence interval, Results that do not overlap with zero are considered statistically significant. Lower HADS scores signify better outcomes. Negative values indicate that column therapy is superior to row therapy. The results derived from the fixed-effects models.

**Capsaicin (SUCRA 90.1%)**						
−0.08 (−0.94, 0.77)	**2–5%THC (SUCRA 89.9%)**					
−0.47 (−1.33, 0.38)	−0.39 (−0.82, 0.04)	**Cannabis (SUCRA 69.4%)**				
**−1.44 (-2.52, -0.36)**	−1.27 (−3.25, 0.70)	−1.36 (−2.25, 0.46)	**Curcumin (SUCRA 39.8%)**			
**−1.50 (-2.55, -0.45)**	**−1.42 (-2.27, -0.56)**	−1.03 (−1.88, 0.17)	−0.06 (−1.14, 1.02)	**6–9%THC (SUCRA 38.1%)**		
**−1.97 (-3.01, -0.92)**	**−1.88 (-2.73, -1.03)**	**−1.49 (-2.35, 0.65)**	−0.53 (−1.60, 0.55)	−0.47 (−1.51, 0.58)	**VitaminD (SUCRA 22.3%)**	
**−2.70 (-3.45, -1.96)**	**−2.62 (-3.04, -2.19)**	**−2.23 (-2.65, -1.80)**	**−1.26 (-2.05, -0.47)**	**−1.20 (-1.95, -0.46)**	**−0.74 (-1.47,0.00)**	**Placebo (SUCRA 0.4%)**

Bold font represents statistical difference.

### Patient global impression of change (PGIC)

The network meta-evidence diagram is shown in Figure 6A. All *p*-values for the indirect and direct comparisons between studies were exceeded 0.05, indicating that the consistency of effects among the studies is acceptable. Further details can be found in Supplementary Table S7.

The NMA results indicated that in improving PGIC scores, 2–5%THC (MD = −1.59, 95% CI: -2.21 to −0.97) and Cannabis (MD = −0.64, 95% CI: -1.14 to −0.14) were superior to placebo treatment. Details are provided in Table 5. Regarding probability rankings among different bioactive compounds for improving PGIC scores, 2–5%THC ranked first in SUCRA (SUCRA = 84.8%, as shown in Figure 6B).

**TABLE 5:** Treatments ranked by SUCRA values and the league table of their relative effectiveness in terms of PGIC. Treatments are ordered by SUCRA values (higher percentage indicates greater probability of being effective). The league table presents pairwise comparisons as mean differences with 95% confidence interval, Results that do not overlap with zero are considered statistically significant. Lower PGIC scores signify better outcomes. Negative values indicate that column therapy is superior to row therapy. The results derived from the random-effects models.

**2–5%THC (SUCRA 85.1%)**								
0.26 (−1.3, 1.82)	**6–9%THC (SUCRA 79.6%)**							
0.11 (−0.94,1.16)	0.84 (−1.23, 2.91)	**Cannabis (SUCRA 72.8%)**						
0.52 (−1.58, 3.61)	0.25 (−1.09,1.59)	0.41 (−1.67, 2.49)	**Curcumin (SUCRA 63.1%)**					
0.54 (−1.59, 3.67)	0.28 (−1.08, 1.63)	0.44 (−1.68, 2.56)	0.02 (−1.06, 1.11)	**Capsaicin (SUCRA 38.4%)**				
0.76 (−1.83, 3.34)	0.49 (−1.53, 2.51)	0.65 (−1.92, 3.22)	0.24 (−1.15, 1.63)	0.22 (−1.20, 1.63)	**Usual treatment (SUCRA 34.5%)**			
0.97 (−1.59,3.54)	0.71 (−1.33, 2.75)	0.87 (−1.68, 3.55)	0.46 (−1.06, 1.97)	0.43 (−1.09, 1.95)	0.22 (−1.53, 1.97)	**VitaminD (SUCRA 14.4%)**		
−1.27 (−3.25, 0.70)	0.38 (−1.01, 1.77)	0.54 (−2.00, 3.07)	0.12 (−1.67, 1.91)	1.10 (−1.67, 3.87)	0.89 (−1.87, 3.65)	0.67 (−1.64, 2.98)	**Alpha-lipoic acid (SUCRA 10.9%)**	
**−1.59 (-2.21, -0.97)**	0.77 (−1.94, 3.48)	**−0.64 (-1.14, -0.14)**	1.52 (−1.00, 4.04)	1.50 (−1.06, 4.06)	1.28 (−1.79, 4.36)	1.06 (−1.99, 4.12)	0.40 (−1.47, 2.27)	**Placebo (SUCRA 0.4%)**

Bold font represents statistical difference.

### Leeds sleep evaluation questionnaire (LSEQ)

The network meta-evidence diagram for LSEQ was presented in Figure 7A. The consistency tests between indirect and direct comparisons among all studies showed that the p-values were greater than 0.05, indicating an acceptable level of consistency. Further detailed information is provided in Supplementary Table S8.

The NMA results suggested that while there were differences in probability rankings among bioactive compounds, none demonstrated a statistically significant improvement in LSEQ scores over conventional measures based on the effect estimates. 2%–5% THC had the highest probability of being the most effective bioactive compound (SUCRA = 90.7%, as shown in Figure 7B)), with an effect size versus placebo of (MD = 0.48, 95% CI: -0.55–1.51). However, the wide confidence interval intersecting the null value and the lack of a significant difference from conventional measures indicate that this finding is uncertain and should be interpreted with caution. Table 6 provides a comparison of different intervention measures.

**TABLE 6:** Treatments ranked by SUCRA values and the league table of their relative effectiveness in terms of LSEQ. Treatments are ordered by SUCRA values (higher percentage indicates greater probability of being effective). The league table presents pairwise comparisons as mean differences with 95% confidence interval, Results that do not overlap with zero are considered statistically significant. Higher LSEQ scores signify better outcomes. Positive values indicate that column therapy is superior to row therapy. The results derived from the fixed-effects models.

**2–5%THC (SUCRA 90.7%)**			
0.59 (−0.59,1.77)	**Cannabis (SUCRA 59.3%)**		
0.79 (−0.39,1.97)	0.20 (−0.66,1.06)	**Placebo (SUCRA 40.7%)**	
1.27 (−0.12,2.65)	0.68 (−0.18,1.54)	0.48 (−0.55,1.51)	**6–9%THC (SUCRA 9.3%)**

### Adverse effects

Adverse reactions associated with cannabinoids and capsaicin were reported across most of the included trials. Among cannabinoids, the most frequently reported adverse events included headache, dry eyes, burning sensation, dizziness, nausea and vomiting, cough, and euphoria. Using the RR as the outcome measure, corresponding statistical analyses were conducted. The p-values for dizziness and euphoria were 0.0002 and 0.02, respectively, indicating statistically significant differences. For capsaicin, commonly reported adverse reactions primarily included erythema, headache, pruritus, burning sensation, and tearing eyes. Detailed information can be found in Table 7.

**TABLE 7:** Summary of adverse effect.

Cannabinoids-related adverse effect	Number of studies included	Exp n/(N) (%)	Con n/N (%)	Risk ratio (95% Cl)	p-value for statistical significance	p-value for heterogeneity
headache	2	8/53	6/56	1.47 [0.42, 5.18]	0.49	0.89
dry eyes	1	1/21	0/22	3.29 [0.13, 85.44]	0.47	-
burning sensation	4	10/135	8/136	1.28 [0.49, 3.38]	0.62	0.98
dizziness	3	33/101	11/104	4.62 [2.07, 10.30]	0.0002**	0.87
nauseaand vomiting	3	12/93	6/96	2.24 [0.79, 6.31]	0.13	0.86
cough	1	3/21	1/22	3.50 [0.33, 36.67]	0.30	-
euphoria	3	22/69	8/72	6.93 [1.32, 36.29]	0.02*	0.27

Capsaicin-related adverse effect	Number of studies included	Exp n/(N) (%)	Con n/N (%)	Risk ratio (95% Cl)	p-value for statistical significance	p-value for heterogeneity
Erythema	3	38/366	6/221	2.81 [0.50, 15.92]	0.24	0.12
headache	1	1/290	0/130	1.35 [0.05, 33.42]	0.85	-
pruritus	4	29/377	8/232	5.37 [2.29, 12.57]	<0.0001***	0.71
Burning	3	24/87	5/102	7.32 [2.64, 20.32]	<0.0001***	0.80
tearing eyes	2	4/76	1/91	3.36 [0.50, 22.73]	0.21	0.53

*P < 0.05; **P < 0.001; ***P < 0.0001.

Abbreviations: N, number; Exp, Experimental; Con, Control.

### Assessment of heterogeneity and inconsistency

Meta-analysis using a random-effects model revealed considerable heterogeneity (I^2^ > 50%) for VAS, NPS, and PGIC outcomes, whereas heterogeneity was low for HADS and LSEQ. Therefore, subgroup analyses were performed to explore sources of heterogeneity based on the route of administration, pain etiology and intervention time, as shown in Table 8.

**TABLE 8:** Summary of subgroup analysis of the route of administration, pain etiology and intervention time on primary outcomes.

Primary outcome	Subgroup	Number of studies	Experimental (N)	Control (N)	Weighed mean (95% Cl)	p-value For heterogeneity	I^2^ test for heterogeneity (%)
VAS	Route of administration						
	Inhaled	8	244	245	−1.08 (−1.34, −0.82)	0.57	0
	Topical	5	630	467	−1.55 (−1.70, −1.41)	0.03*	76
	Oral	1	165	165	−1.00 (−1.44, −0.56)	-	-
	Pain etiology						
	Diabetic	5	268	283	−1.42 (−2.08, −0.76)	0.06	46
	Peripheral	4	580	401	−1.11 (−1.83, −0.39)	0.85	0
	Central	5	191	191	−1.35 (−1.86, −0.85)	0.02*	65
	Intervention time						
	<8 weeks	6	175	174	−1.13(−1.49, −0.77)	0.04*	57
	8–12 weeks	7	699	536	−1.39(−1.98, −0.80)	0.002*	84
	>12 weeks	1	165	165	−0.62(−0.84, −0.40)	—	—
NPS	Route of administration						
	Inhaled	6	206	206	−0.93 (−1.15, −0.70)	0.18	40
	Oral	5	359	352	−2.50 (−2.76, −2.24)	<0.001**	80
	Pain etiology						
	Diabetic	5	359	352	−3.08 (−3.64, −2.52)	0.04*	64
	Peripheral	1	15	15	−0.88 (−1.99, −0.23)	—	—
	Central	5	191	191	−0.90 (−1.26, −0.54)	0.20	38
	Intervention time						
	<8 weeks	6	206	206	−0.88 (−1.12, −0.65)	0.0002**	82
	8–12 weeks	3	237	230	−2.22 (−2.52, −1.91)	0.21	45
	>12 weeks	2	122	122	−1.57 (−1.86, −1.28)	0.03*	69
PGIC	Route of administration						
	Inhaled	6	497	336	−1.35 (−1.58, −1.11)	0.19	39
	Topical	2	301	141	−1.55 (−1.92, −1.18)	0.004*	88
	Oral	3	162	162	−1.07 (−1.43, −0.72)	0.92	0
	Pain etiology						
	Diabetic	5	521	345	−0.76 (−1.43, −0.10)	0.08	55
	Peripheral	2	312	151	−1.25 (−2.25, −0.25)	0.02*	82
	Central	4	127	143	−1.38 (−0.86, −0.10)	0.76	0
	Intervention time						
	<8 weeks	4	196	195	−1.19 (−1.37, −1.02)	0.68	0
	8–12 weeks	5	703	383	−1.37 (−1.60, −1.14)	0.92	0
	>12 weeks	2	61	61	−0.95 (−1.28, −0.63)	0.72	0

*P < 0.05; **P < 0.001.

Abbreviations: N, number; VAS, visual analogue scale; NPS, neuropathic pain scale; PGIC, patient global impression of change.

Analysis by route of administration suggested that inhaled administration might be a source of heterogeneity for VAS and NPS, while topical and oral administration contributed to heterogeneity in VAS and PGIC, respectively. Subgrouping by pain etiology indicated that peripheral neuropathy may explain heterogeneity in VAS, whereas central neuropathy was associated with heterogeneity in NPS and PGIC. Analysis by intervention duration showed that time frame was a significant source of heterogeneity only for PGIC, with the strongest treatment effect observed in the medium-term (8–12 weeks).

Inconsistency assessment showed no significant global inconsistency for VAS, HADS, PGIC, or LSEQ (all p > 0.05). Node-splitting analysis confirmed local consistency for VAS and PGIC, indicating that their high heterogeneity likely stemmed from clinical or methodological diversity rather than statistical inconsistency. In contrast, global inconsistency was detected for NPS (p < 0.05), which was localized to the comparison between 2% and 5% THC and placebo, possibly due to differences in dosing or trial design between direct and indirect evidence. The results were summarized in the Supplementary Tables S9–S13.

### Sensitivity analysis results

Sensitivity analyses were conducted to assess the robustness of the primary NMA findings. In the first analysis, studies at high risk of bias, with imputed data, digitally extracted data, or employing vague unit scales were sequentially excluded. The results remained consistent, with the estimated effect sizes, confidence intervals, and statistical significance of the primary endpoints unchanged. This indicates that the primary conclusions are robust against methodological variations.

A second sensitivity analysis was conducted to address the clinical heterogeneity in vitamin D administration routes, which included both oral and intramuscular (IM) forms. The network was restructured by dividing vitamin D into two nodes (vitamin D _oral and vitamin D _ IM) (Supplementary Figures S6, S7) while keeping all other interventions unchanged. The NMA was re-run using the same statistical model and prior distributions as the primary analysis. The results showed good global and local consistency, and the relative treatment effects for all other interventions in the network were not significantly altered (Supplementary Tables S14, S15). This confirms that the variation in vitamin D administration did not significantly impact the overall findings, reinforcing the reliability of our conclusions.

### The risk of publication bias

**FIGURE 8:**
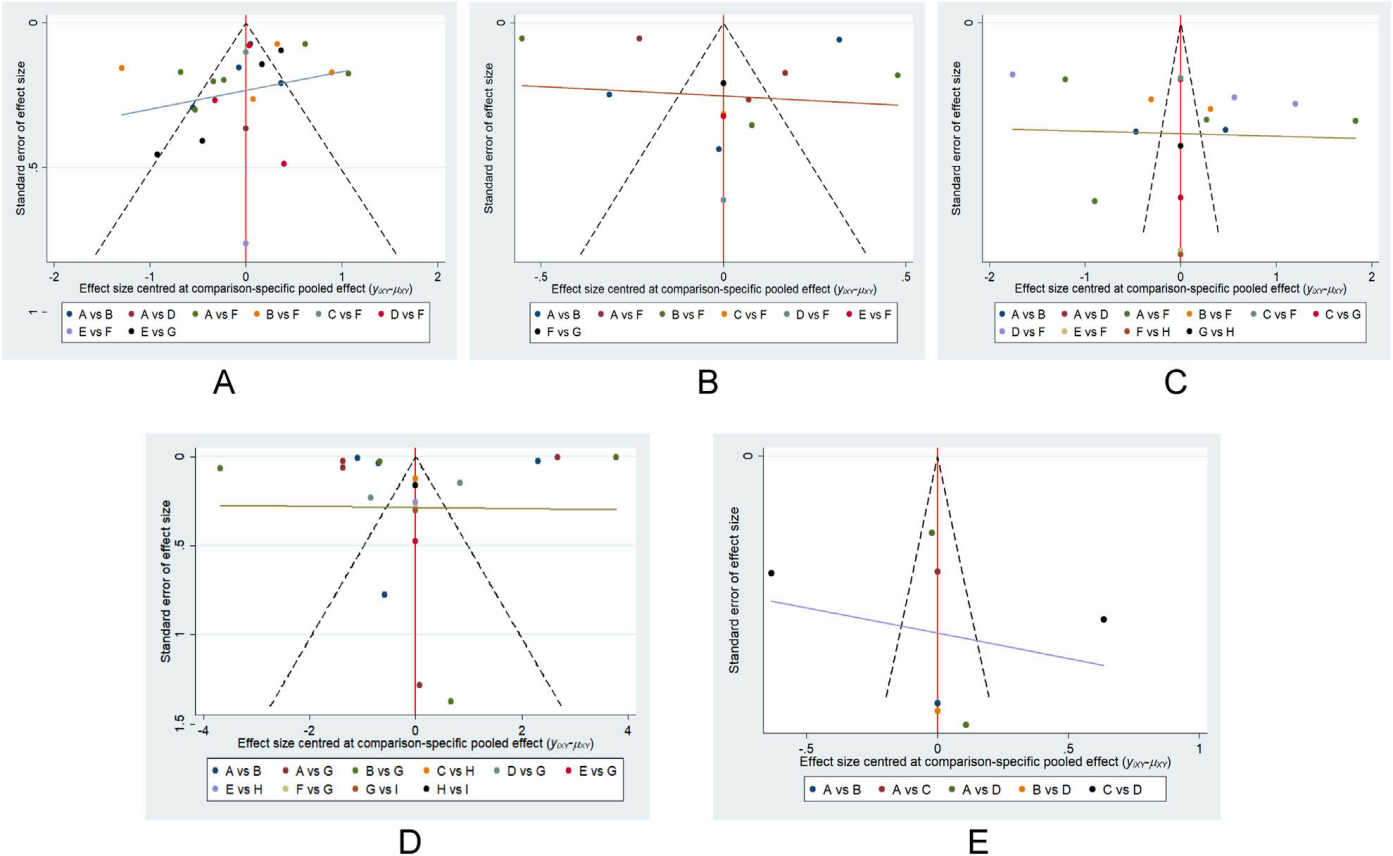
Funnel plot on publication bias. **(A)** VAS; **(B)** NPS; **(C)** HADS; **(D)** PGIC; **(E)** LSEQ.

We conducted an assessment of potential publication bias through the construction of separate funnel plots for each outcome result. Upon visual inspection of these funnel plots, no substantial evidence of bias was detected (Wallace et al., 2009). Further details are presented in Figure 8. This subjective impression was confirmed by Egger’s linear regression test, which found no statistically significant evidence of funnel plot asymmetry (Supplementary Table S16).

### GRADE rating of outcome indicators

**FIGURE 9:**
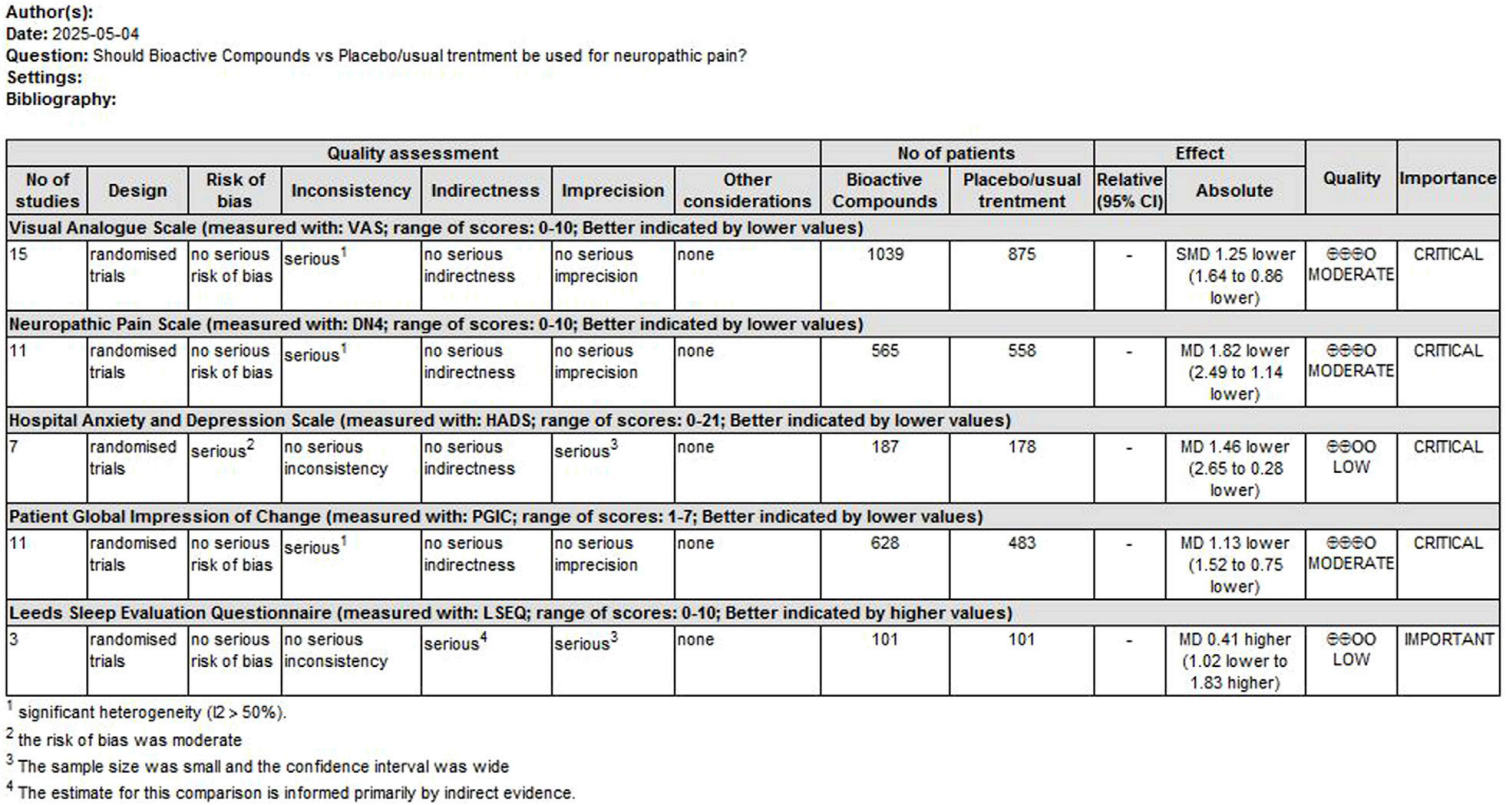
GRADE rating for evidence of primary outcomes. Abbreviations: SMD, standardized mean difference; MD, mean difference.

GRADE rating was performed for primary outcomes. We found that the evidence levels of primary outcomes were rated as moderate to low. Mainly because the risks of bias, inconsistency, imprecision and indirection downgraded the overall quality assessment (Figure 9).

GRADE downgrade reasons including: (1) Risk of Bia; We downgraded by one level due to serious concerns regarding risk of bias across studies. Trials had high/unclear risk in key domains (randomization, blinding, incomplete outcome data) potentially biasing effect estimates. (2) Inconsistency (Heterogeneity); Evidence was downgraded by one level for unexplained heterogeneity (I^2^ >50%, p < 0.05). (3) Imprecision; We downgraded by one level for imprecision. The confidence interval crossed the predefined minimal clinically important difference threshold of and/or included both clinically significant benefit and no effect. (4) Indirectness: We downgraded by one level due to the lack of a formal transitivity assessment, which has introduced uncertainty in indirect comparisons.

## Discussion

The main aim of this NMA was to evaluate the efficacy of different bioactive compounds on pain intensity and quality of life in NP patients. A total of 20 RCTs were included, covering 6 different natural products and involving a large sample of 2471 patients diagnosed with NP. While the NMA yielded probability rankings that favored THC for pain intensity, 2%–5% THC and capsaicin for certain quality-of-life measures, the clinical implications of these findings are uncertain. The evidence for these rankings should be interpreted with caution due to the precision of effect and the need to reference MCIDs.

Our study suggested that 6%–9% THC exhibited advantages in reducing VAS scores, which is supported by its mechanism of action. Neuroimaging evidence indicates that THC may alleviate pain by disrupting functional connectivity in pain-processing pathways like the anterior cingulate cortex (ACC)—a region rich in cannabinoid receptors (Weizman et al., 2018; Lee et al., 2013; Eggan and Lewis, 2007). Although the point estimates suggested a potential benefit of 6%–9% THC over capsaicin (−0.28) and alpha-lipoic acid (−0.67), the differences did not reach statistical significance. It is critical to note that this lack of statistical significance does not equate to evidence of no difference. Given that the evidence for this comparison was rated as moderate certainty and this observed difference fell below the established MCID of 2 points. Therefore, we can be reasonably confident that 6%–9% THC does not confer a clinically meaningful advantage over capsaicin or alpha-lipoic acid, despite its superior ranking. Thus, the choice between these interventions may depend on factors such as safety, cost, and patient preference rather than efficacy. Furthermore, the therapeutic potential of THC is tempered by a considerable risk of adverse events. Our findings confirm that dizziness and euphoria are significant concerns, which aligns with broader evidence of dose-related cognitive and psychiatric effects (Whiting et al., 2015; Pope et al., 2001; Hart et al., 2001). Clinicians must carefully consider this risk-benefit profile before prescribing.

Our NMA also indicated that 2%–5% THC had the highest probability of effective intervention for reducing NPS. However, the lack of a statistically significant difference from the next-best options (6–9%THC and alpha-lipoic acid), supported by moderate-certainty evidence, implies a comparable analgesic effect among them. Therefore, the critical differentiating factor becomes safety. Existing evidence clearly indicated that a lower dose of THC was effective while having a significantly lesser impact on cognition compared to the higher dose (Wilsey et al., 2008). Therefore, lower doses may offer a superior risk-benefit profile by balancing analgesia with reduced neurocognitive adverse events. This approach is particularly relevant in the context of polypharmacy for chronic pain, where adding a low-dose THC could be a strategy for treatment-resistant cases without exacerbating side effects (Black and Sang, 2005; Cichewicz, 2004).

This analysis proposed that capsaicin might offer a dual benefit in neuropathic pain by improving both pain intensity (VAS) and emotional distress (HADS). Mechanistically, this effect is attributed to capsaicin’s agonist action on transient receptor potential vanilloid-1 (TRPV1) receptors, which initially causes transient burning or stinging due to nociceptor activation, followed by prolonged desensitization (often termed “de-functionalization”) that underlies its sustained analgesic effect (Groninger and Schisler, 2012; Dessirier et al., 2000). Although capsaicin was ranked highest, the differences from subsequent interventions were not statistically significant. The evidence supporting this comparison was of low certainty, due to risk of bias and imprecision in the included studies, means we have little confidence in the estimated ranking. Therefore, the current evidence is insufficient to reliably distinguish the effects of these interventions on emotional outcomes. In terms of safety, the primary concern remains the self-limiting, initial local irritation, while other reactions such as erythema may occur (Parrott and Hindmarch, 1980). In summary, while capsaicin remains a useful non-systemic option in selected patients, the current evidence is insufficient to robustly support its superiority in improving emotional symptoms over other treatments.

Although our analysis suggested that low-dose (2%–5%) THC had the highest probability of being the most effective intervention for improving patient-reported outcomes on the PGIC and LSEQ scales, this finding must be interpreted with caution for several key reasons. First, the certainty of the evidence supporting these rankings was not uniform across outcomes. For the improvement in PGIC, the evidence was rated as moderate, indicating that the true effect is likely close to the estimated one. However, for LSEQ, the evidence was downgraded to low certainty, meaning our confidence in the effect estimate is limited. Consequently, the conclusion regarding sleep improvement is considerably more uncertain. Second, and most critically, the clinical relevance of this statistical superiority is questionable. When the estimated benefits were evaluated against the MCID, low-dose THC was superior only to placebo and alpha-lipoic acid, but not to other active comparators. This indicates that while the effect of low-dose THC may be statistically detectable, its clinical advantage over a range of alternative treatments remains uncertain. Overall, the current evidence is insufficient to robustly recommend low-dose THC over other therapeutic options. Any potential benefit must be carefully weighed against its known adverse effects, and shared decision-making with patients is essential.

Furthermore, our research confirmed that alpha-lipoic acid was superior to placebo in improving VAS and NPS scores. As a mitochondrial coenzyme used in oxidative stress-related conditions such as diabetes and neurodegenerative diseases (Packer et al., 1995), Alpha-lipoic acid at 600 mg/day significantly improved symptom scores and remission rates in diabetic neuropathy patients, with similar benefits observed in back and neck pain studies, likely due to its antioxidant effect (Ziegler et al., 2004; Ranieri et al., 2009; Letizia Mauro et al., 2014). Additionally, curcumin showed advantage in alleviating HADS scores in neuropathic pain patients, consistent with its reported efficacy in reducing depression and anxiety in diabetic neuropathy (Asadi et al., 2019). Its antidepressant and anxiolytic effects are linked to anti-inflammatory, antioxidant, and neurotransmitter-modulating properties (Lopresti et al., 2012; Benammi et al., 2014). However, the poor oral bioavailability of curcumin in human causes a major obstacle to achieving adequate plasma levels with desirable pharmacological effects. It is preferable to use Nano-curcumin, which shows more efficacy and faster cellular absorption than free curcumin (Rahimi et al., 2016). Consequently, in both of the studies that we incorporated, nano-curcumin supplementation was utilized instead of free curcumin.

Our meta-analysis revealed considerable heterogeneity for several primary outcomes, notably VAS, NPS, and PGIC. However, for VAS and PGIC, the absence of inconsistency indicates that the heterogeneity likely stems from clinical diversity within comparisons (e.g., patient populations, intervention protocols) rather than statistical conflicts between direct and indirect evidence. Subgroup analyses suggested that the route of administration, pain etiology, and intervention time are potential effect modifiers. A notable time-dependent effect was found for PGIC, with the greatest benefit observed in the medium term (8–12 weeks). In contrast, the NPS outcome showed significant global inconsistency, specifically in the 2%–5% THC vs. placebo comparison. This suggests a conflict between direct and indirect evidence, possibly due to differences in trial design or populations, necessitating cautious interpretation of NPS results. In summary, the treatment effects are influenced by key clinical and methodological variables. The coherent networks for VAS and PGIC support their robust efficacy signals, despite heterogeneity. The inconsistent NPS network highlights a more complex evidence base. Future trials should standardize protocols and target specific patient subgroups to clarify these effects.

The sensitivity analyses conducted in this study strengthen the credibility of our primary NMA findings. The consistency of the results after the sequential exclusion of studies with potential methodological concerns demonstrates that the core conclusions are robust against these sources of bias. This robustness enhances our confidence that the observed treatment effects are not merely artifacts of specific methodological choices or data quality limitations within the included trials. Furthermore, the second sensitivity analysis, which explicitly modeled the different administration routes of vitamin D (oral and intramuscular), confirmed that this specific clinical heterogeneity did not materially influence the network estimates. The preservation of both global and local consistency, along with stable effect estimates for all other interventions, indicates that the network structure was resilient to this modification. This finding is crucial, as it suggests that the relative ranking of treatments is reliable despite variations in how certain interventions are delivered in clinical practice. Collectively, these analyses affirm the stability and clinical applicability of our results, supporting their potential utility in informing treatment decisions for neuropathic pain.

## Limitations

Our research also has some inevitable limitations: (1) The results of our study had significant heterogeneity in the primary outcomes, and the source of heterogeneity could not be completely determined by subgroup analysis because of the limited data provided by the original trials, our findings must be treated with caution. (2) The risk of bias in existing studies, the heterogeneity of results, and the resulting high degree of uncertainty were the main reasons for downgrading the strength of evidence for some outcomes. The current recommendations are based on the above-mentioned trade-offs and strongly rely on the results of future high-quality research. (3) QoL was assessed via patient-reported questionnaires; functional outcomes (e.g., work absenteeism, objective activity monitoring, or analgesic consumption) were not captured. While this aligns with our focus on subjective patient experience, future studies should include these measures to comprehensively evaluate treatment efficacy. (4) The lack of pain duration, detailed data on comorbidities and concurrent treatments, the generalization of the research results needs to be cautious, especially when it comes to patient groups with different pain characteristics, comorbidities or receiving specific combined treatments. (5) The limited number of studies considered in our research and the lack of direct comparative evidence for certain intervention measures, these results should be interpreted with caution. (6) The absence of a formal transitivity assessment is also a limitation. Clinical heterogeneity could bias indirect estimates, necessitating cautious interpretation, particularly for comparisons based on indirect evidence. This was considered in our GRADE ratings by downgrading for indirectness.

## Conclusion

The results of the NMA show that bioactive compounds have respective advantages in improving the pain intensity and quality of life of patients with neuropathic pain. This conclusion is supported by multiple sensitivity analyses. However, higher-certainty evidence is needed for universal recommendation. In addition, due to the limitations of the initial research design, the comparisons of certain treatment methods were indirect, which might have weakened the strength of the evidence in our study. Therefore, there is an urgent need to conduct more rigorous and high-quality randomized controlled trials to further verify the efficacy and safety of bioactive compounds in the treatment of neuropathic pain.

## Data Availability

The original contributions presented in the study are included in the article/Supplementary Material, further inquiries can be directed to the corresponding authors.

## References

[B1] Abbas W. Alam Khan R. Tasawar Baig M. Shaikh S. A. (2022). Effect of Curcuma longa on glycemia, neuropathic sensation and advanced glycation end product in diabetic patients. Pak. J. Pharm. Sci. 35 (3(Special)), 873–878. 35791581

[B2] Agathos E. Tentolouris A. Eleftheriadou I. Katsaouni P. Nemtzas I. Petrou A. (2018). Effect of α-lipoic acid on symptoms and quality of life in patients with painful diabetic neuropathy. J. Int. Med. Res. 46 (5), 1779–1790. 10.1177/0300060518756540 29517942 PMC5991249

[B3] Agoons B. B. Dehayem Yefou M. Katte J. C. Etoa Etoga M. C. Agoons D. D. Yepnjio F. (2020). Effect of topical capsaicin on painful sensory peripheral neuropathy in patients with type 2 diabetes: a double-blind placebo-controlled randomised clinical trial. Cureus 12 (10), e11147. 10.7759/cureus.11147 33251057 PMC7685815

[B4] Asadi S. Gholami M. S. Siassi F. Qorbani M. Khamoshian K. Sotoudeh G. (2019). Nano curcumin supplementation reduced the severity of diabetic sensorimotor polyneuropathy in patients with type 2 diabetes mellitus: a randomized double-blind placebo-controlled clinical trial. Complement. Ther. Med. 43, 253–260. 10.1016/j.ctim.2019.02.014 30935539

[B5] Benammi H. El Hiba O. Romane A. Gamrani H. (2014). A blunted anxiolytic like effect of curcumin against acute lead induced anxiety in rat: involvement of serotonin. Acta histochem. 116 (5), 920–925. 10.1016/j.acthis.2014.03.002 24721902

[B6] Berman J. S. Symonds C. Birch R. (2004). Efficacy of two cannabis based medicinal extracts for relief of central neuropathic pain from brachial plexus avulsion: results of a randomised controlled trial. Pain 112 (3), 299–306. 10.1016/j.pain.2004.09.013 15561385

[B7] Black D. R. Sang C. N. (2005). Advances and limitations in the evaluation of analgesic combination therapy. Neurology 65 (12 Suppl. 4), S3–S6. 10.1212/wnl.65.12_suppl_4.s3 16385102

[B8] Blake D. R. Robson P. Ho M. Jubb R. W. McCabe C. S. (2006). Preliminary assessment of the efficacy, tolerability and safety of a cannabis-based medicine (sativex) in the treatment of pain caused by rheumatoid arthritis. Rheumatol. Oxf. 45 (1), 50–52. 10.1093/rheumatology/kei183 16282192

[B9] Chaimani A. Higgins J. P. T. Mavridis D. Spyridonos P. Salanti G. (2013). Graphical tools for network meta-analysis in STATA. PLoS One 8 (10), e76654. 10.1371/journal.pone.0076654 24098547 PMC3789683

[B10] Cichewicz D. L. (2004). Synergistic interactions between cannabinoid and opioid analgesics. Life Sci. 74 (11), 1317–1324. 10.1016/j.lfs.2003.09.038 14706563

[B11] Colloca L. Ludman T. Bouhassira D. Baron R. Dickenson A. H. Yarnitsky D. (2017). Neuropathic pain. Nat. Rev. Dis. Prim. 3, 17002. 10.1038/nrdp.2017.2 28205574 PMC5371025

[B12] Cruccu G. Nurmikko T. J. Ernault E. Riaz F. K. McBride W. T. Haanpää M. (2018). Superiority of capsaicin 8% patch *versus* oral pregabalin on dynamic mechanical allodynia in patients with peripheral neuropathic pain. Eur. J. Pain 22 (4), 700–706. 10.1002/ejp.1155 29194851 PMC5887877

[B13] Cumpston M. Li T. Page M. J. Chandler J. Welch V. A. Higgins J. P. (2019). Updated guidance for trusted systematic reviews: a new edition of the cochrane handbook for systematic reviews of interventions. Cochrane Database Syst. Rev. 10 (10), Ed000142. 10.1002/14651858.ED000142 31643080 PMC10284251

[B14] Dessirier J. M. Simons C. T. Sudo M. Sudo S. Carstens E. (2000). Sensitization, desensitization and stimulus-induced recovery of trigeminal neuronal responses to oral capsaicin and nicotine. J. Neurophysiol. 84 (4), 1851–1862. 10.1152/jn.2000.84.4.1851 11024077

[B15] Dworkin R. H. Turk D. C. Wyrwich K. W. Beaton D. Cleeland C. S. Farrar J. T. (2008). Interpreting the clinical importance of treatment outcomes in chronic pain clinical trials: IMMPACT recommendations. J. Pain 9 (2), 105–121. 10.1016/j.jpain.2007.09.005 18055266

[B16] Eggan S. M. Lewis D. A. (2007). Immunocytochemical distribution of the cannabinoid CB1 receptor in the primate neocortex: a regional and laminar analysis. Cereb. Cortex 17 (1), 175–191. 10.1093/cercor/bhj136 16467563

[B17] Evans J. L. Goldfine I. D. (2000). Alpha-lipoic acid: a multifunctional antioxidant that improves insulin sensitivity in patients with type 2 diabetes. Diabetes Technol. Ther. 2 (3), 401–413. 10.1089/15209150050194279 11467343

[B18] Finnerup N. B. Attal N. Haroutounian S. McNicol E. Baron R. Dworkin R. H. (2015). Pharmacotherapy for neuropathic pain in adults: a systematic review and meta-analysis. Lancet Neurol. 14 (2), 162–173. 10.1016/S1474-4422(14)70251-0 25575710 PMC4493167

[B19] Gaskin D. J. Richard P. (2012). The economic costs of pain in the United States. J. Pain 13 (8), 715–724. 10.1016/j.jpain.2012.03.009 22607834

[B20] Gilron I. Baron R. Jensen T. (2015). Neuropathic pain: principles of diagnosis and treatment. Mayo Clin. Proc. 90 (4), 532–545. 10.1016/j.mayocp.2015.01.018 25841257

[B21] Groninger H. Schisler R. E. (2012). Topical capsaicin for neuropathic pain #255. J. Palliat. Med. 15 (8), 946–947. 10.1089/jpm.2012.9571 22849599 PMC3462404

[B22] Guyatt G. Oxman A. D. Akl E. A. Kunz R. Vist G. Brozek J. (2011). GRADE guidelines: 1. Introduction-GRADE evidence profiles and summary of findings tables. J. Clin. Epidemiol. 64 (4), 383–394. 10.1016/j.jclinepi.2010.04.026 21195583

[B23] Hart C. L. van Gorp W. Haney M. Foltin R. W. Fischman M. W. (2001). Effects of acute smoked marijuana on complex cognitive performance. Neuropsychopharmacology 25 (5), 757–765. 10.1016/S0893-133X(01)00273-1 11682259

[B24] Higgins J. P. Altman D. G. Gøtzsche P. C. Jüni P. Moher D. Oxman A. D. (2011). The cochrane Collaboration's tool for assessing risk of bias in randomised trials. Bmj 343, d5928. 10.1136/bmj.d5928 22008217 PMC3196245

[B25] Hozo S. P. Djulbegovic B. Hozo I. (2005). Estimating the mean and variance from the median, range, and the size of a sample. BMC Med. Res. Methodol. 5, 13. 10.1186/1471-2288-5-13 15840177 PMC1097734

[B26] Jensen T. S. Baron R. Haanpää M. Kalso E. Loeser J. D. Rice A. S. C. (2011). A new definition of neuropathic pain. Pain 152 (10), 2204–2205. 10.1016/j.pain.2011.06.017 21764514

[B27] Kiani J. Sajedi F. Nasrollahi S. A. Esna-Ashari F. (2015). A randomized clinical trial of efficacy and safety of the topical clonidine and capsaicin in the treatment of painful diabetic neuropathy. J. Res. Med. Sci. 20 (4), 359–363. 10.4103/1735-1995.158258 26109991 PMC4468451

[B28] Lee M. C. Ploner M. Wiech K. Bingel U. Wanigasekera V. Brooks J. (2013). Amygdala activity contributes to the dissociative effect of cannabis on pain perception. Pain 154 (1), 124–134. 10.1016/j.pain.2012.09.017 23273106 PMC3549497

[B29] Letizia Mauro G. Cataldo P. Barbera G. Sanfilippo A. (2014). α-Lipoic acid and superoxide dismutase in the management of chronic neck pain: a prospective randomized study. Drugs R. D. 14 (1), 1–7. 10.1007/s40268-013-0035-3 24477618 PMC3964291

[B30] Lopresti A. L. Hood S. D. Drummond P. D. (2012). Multiple antidepressant potential modes of action of curcumin: a review of its anti-inflammatory, monoaminergic, antioxidant, immune-modulating and neuroprotective effects. J. Psychopharmacol. 26 (12), 1512–1524. 10.1177/0269881112458732 23035031

[B31] Mankowski C. Poole C. D. Ernault E. Thomas R. Berni E. Currie C. J. (2017). Effectiveness of the capsaicin 8% patch in the management of peripheral neuropathic pain in European clinical practice: the ASCEND study. BMC Neurol. 17 (1), 80. 10.1186/s12883-017-0836-z 28431564 PMC5399813

[B32] Mehta S. Nain P. Agrawal B. K. Singh R. P. Kaur J. Maity S. (2021). Effectiveness of empagliflozin with vitamin D supplementation in peripheral neuropathy in type 2 diabetic patients. Cureus J. Med. Sci. 13 (12), e20208. 10.7759/cureus.20208 35004028 PMC8730350

[B33] Mendoza C. Kraemer P. Herrera P. Burdiles P. Sepúlveda D. Núñez E. (2017). Clinical guidelines using the GRADE system (grading of recommendations assessment, development and evaluation). Rev. Med. Chil. 145 (11), 1463–1470. 10.4067/s0034-98872017001101463 29664529

[B34] Moher D. Shamseer L. Clarke M. Ghersi D. Liberati A. Petticrew M. (2015). Preferred reporting items for systematic review and meta-analysis protocols (PRISMA-P) 2015 statement. Syst. Rev. 4 (1), 1. 10.1186/2046-4053-4-1 25554246 PMC4320440

[B35] Moulin D. E. Clark A. J. Gilron I. Ware M. A. Watson C. P. N. Sessle B. J. (2007). Pharmacological management of chronic neuropathic pain - consensus statement and guidelines from the Canadian pain society. Pain Res. Manag. 12 (1), 13–21. 10.1155/2007/730785 17372630 PMC2670721

[B36] Nadro B. Lőrincz H. Molnár Á. Szentpéteri A. Zöld E. Seres I. (2021). Effects of alpha-lipoic acid treatment on serum progranulin levels and inflammatory markers in diabetic neuropathy. J. Int. Med. Res. 49 (5), 3000605211012213. 10.1177/03000605211012213 34041950 PMC8165837

[B37] Nurmikko T. J. Serpell M. G. Hoggart B. Toomey P. J. Morlion B. J. Haines D. (2007). Sativex successfully treats neuropathic pain characterised by allodynia: a randomised, double-blind, placebo-controlled clinical trial. Pain 133 (1-3), 210–220. 10.1016/j.pain.2007.08.028 17997224

[B38] Packer L. Witt E. H. Tritschler H. J. (1995). alpha-Lipoic acid as a biological antioxidant. Free Radic. Biol. Med. 19 (2), 227–250. 10.1016/0891-5849(95)00017-r 7649494

[B39] Papaioannou E. (2018). Comparison of capsaicine patch 8% monotherapy and its combination with pregabalin in postherpetic neuralgia treatment. Regional Anesth. pain Med. 43 (7), e181–e182.

[B40] Papanas N. Ziegler D. (2014). Efficacy of α-lipoic acid in diabetic neuropathy. Expert Opin. Pharmacother. 15 (18), 2721–2731. 10.1517/14656566.2014.972935 25381809

[B41] Parrott A. C. Hindmarch I. (1980). The Leeds Sleep evaluation Questionnaire in psychopharmacological investigations - a review. Psychopharmacol. Berl. 71 (2), 173–179. 10.1007/BF00434408 6777817

[B42] Pitcher M. H. Von Korff M. Bushnell M. C. Porter L. (2019). Prevalence and profile of high-impact chronic pain in the United States. J. Pain 20 (2), 146–160. 10.1016/j.jpain.2018.07.006 30096445 PMC8822465

[B43] Pope H. G. Jr. Gruber A. J. Yurgelun-Todd D. (2001). Residual neuropsychologic effects of cannabis. Curr. Psychiatry Rep. 3 (6), 507–512. 10.1007/s11920-001-0045-7 11707165

[B44] Rahimi H. R. Nedaeinia R. Sepehri Shamloo A. Nikdoust S. Kazemi Oskuee R. (2016). Novel delivery system for natural products: Nano-Curcumin formulations. Avicenna J. Phytomed 6 (4), 383–398. 27516979 PMC4967834

[B45] Ranieri M. Sciuscio M. Cortese A. M. Santamato A. Di Teo L. Ianieri G. (2009). The use of alpha-lipoic acid (ALA), gamma linolenic acid (GLA) and rehabilitation in the treatment of back pain: effect on health-related quality of life. Int. J. Immunopathol. Pharmacol. 22 (3 Suppl. l), 45–50. 10.1177/03946320090220S309 19887043

[B46] Rog D. J. Nurmikko T. J. Friede T. Young C. A. (2005). Randomized, controlled trial of cannabis-based medicine in central pain in multiple sclerosis. Neurology 65 (6), 812–819. 10.1212/01.wnl.0000176753.45410.8b 16186518

[B47] Rouse B. Chaimani A. Li T. (2017). Network meta-analysis: an introduction for clinicians. Intern Emerg. Med. 12 (1), 103–111. 10.1007/s11739-016-1583-7 27913917 PMC5247317

[B48] Rowbotham M. Harden N. Stacey B. Bernstein P. Magnus-Miller L. (1998). Gabapentin for the treatment of postherpetic neuralgia: a randomized controlled trial. Jama 280 (21), 1837–1842. 10.1001/jama.280.21.1837 9846778

[B49] Sari A. Akdoğan Altun Z. Arifoglu Karaman C. Bilir Kaya B. Durmus B. (2020). Does vitamin D affect diabetic neuropathic pain and balance? J. Pain Res. 13, 171–179. 10.2147/JPR.S203176 32021406 PMC6970609

[B50] Skrabek R. Q. Galimova L. Ethans K. Perry D. (2008). Nabilone for the treatment of pain in fibromyalgia. J. Pain 9 (2), 164–173. 10.1016/j.jpain.2007.09.002 17974490

[B51] Steingrímsdóttir Ó A. Landmark T. Macfarlane G. J. Nielsen C. S. (2017). Defining chronic pain in epidemiological studies: a systematic review and meta-analysis. Pain 158 (11), 2092–2107. 10.1097/j.pain.0000000000001009 28767506

[B52] Svendsen K. B. Jensen T. S. Bach F. W. (2004). Does the cannabinoid dronabinol reduce central pain in multiple sclerosis? Randomised double blind placebo controlled crossover trial. Bmj 329 (7460), 253. 10.1136/bmj.38149.566979.AE 15258006 PMC498019

[B53] van Hecke O. Austin S. K. Khan R. A. Smith B. H. Torrance N. (2014). Neuropathic pain in the general population: a systematic review of epidemiological studies. Pain 155 (4), 654–662. 10.1016/j.pain.2013.11.013 24291734

[B54] Wallace B. C. Schmid C. H. Lau J. Trikalinos T. A. (2009). Meta-analyst: software for meta-analysis of binary, continuous and diagnostic data. BMC Med. Res. Methodol. 9, 80. 10.1186/1471-2288-9-80 19961608 PMC2795760

[B55] Wallace M. S. Marcotte T. D. Umlauf A. Gouaux B. Atkinson J. H. (2015). Efficacy of inhaled cannabis on painful diabetic neuropathy. J. Pain 16 (7), 616–627. 10.1016/j.jpain.2015.03.008 25843054 PMC5152762

[B56] Ware M. A. Wang T. Shapiro S. Robinson A. Ducruet T. Huynh T. (2010). Smoked cannabis for chronic neuropathic pain: a randomized controlled trial. Cmaj 182 (14), E694–E701. 10.1503/cmaj.091414 20805210 PMC2950205

[B57] Weizman L. Dayan L. Brill S. Nahman-Averbuch H. Hendler T. Jacob G. (2018). Cannabis analgesia in chronic neuropathic pain is associated with altered brain connectivity. Neurology 91 (14), e1285–e1294. 10.1212/WNL.0000000000006293 30185448 PMC6177269

[B58] Whiting P. F. Wolff R. F. Deshpande S. Di Nisio M. Duffy S. Hernandez A. V. (2015). Cannabinoids for medical use: a systematic review and meta-analysis. Jama 313 (24), 2456–2473. 10.1001/jama.2015.6358 26103030

[B59] Wilsey B. Marcotte T. Tsodikov A. Millman J. Bentley H. Gouaux B. (2008). A randomized, placebo-controlled, crossover trial of cannabis cigarettes in neuropathic pain. J. Pain 9 (6), 506–521. 10.1016/j.jpain.2007.12.010 18403272 PMC4968043

[B60] Wilsey B. Marcotte T. Deutsch R. Gouaux B. Sakai S. Donaghe H. (2013). Low-dose vaporized cannabis significantly improves neuropathic pain. J. Pain 14 (2), 136–148. 10.1016/j.jpain.2012.10.009 23237736 PMC3566631

[B61] Wilsey B. Marcotte T. D. Deutsch R. Zhao H. Prasad H. Phan A. (2016). An exploratory human laboratory experiment evaluating vaporized cannabis in the treatment of neuropathic pain from spinal cord injury and disease. J. Pain 17 (9), 982–1000. 10.1016/j.jpain.2016.05.010 27286745 PMC5007175

[B62] Ziegler D. Hanefeld M. Ruhnau K. J. Hasche H. Lobisch M. Schütte K. (1999). Treatment of symptomatic diabetic polyneuropathy with the antioxidant alpha-lipoic acid: a 7-month multicenter randomized controlled trial (ALADIN III study). ALADIN III study group. alpha-lipoic acid in diabetic neuropathy. Diabetes Care 22 (8), 1296–1301. 10.2337/diacare.22.8.1296 10480774

[B63] Ziegler D. Nowak H. Kempler P. Vargha P. Low P. A. (2004). Treatment of symptomatic diabetic polyneuropathy with the antioxidant alpha-lipoic acid: a meta-analysis. Diabet. Med. 21 (2), 114–121. 10.1111/j.1464-5491.2004.01109.x 14984445

