# Supplementary figures and images for: Systematic review and network meta-analysis of the effects of bioactive compounds on pain intensity and quality of life in neuropathic pain patients
Source: Front Pharmacol. 2025 Oct 17;16:1656400. doi: 10.3389/fphar.2025.1656400 (PMC12576042; doi:10.3389/fphar.2025.1656400)

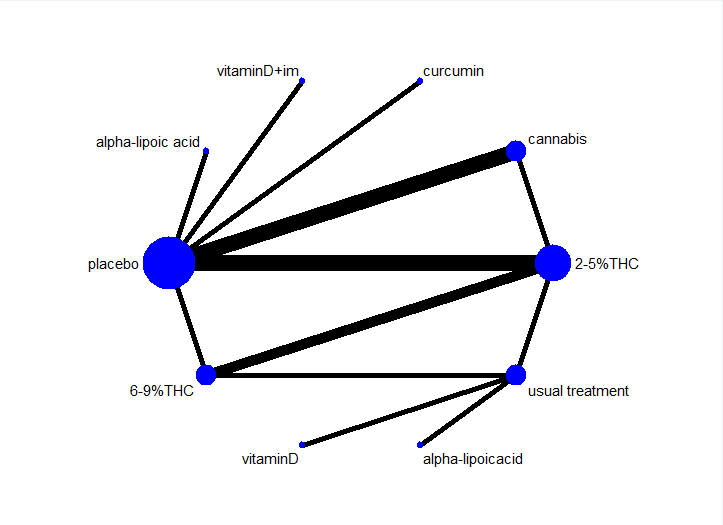

Supplement: Supplementary file 1 [file Image6.tif]

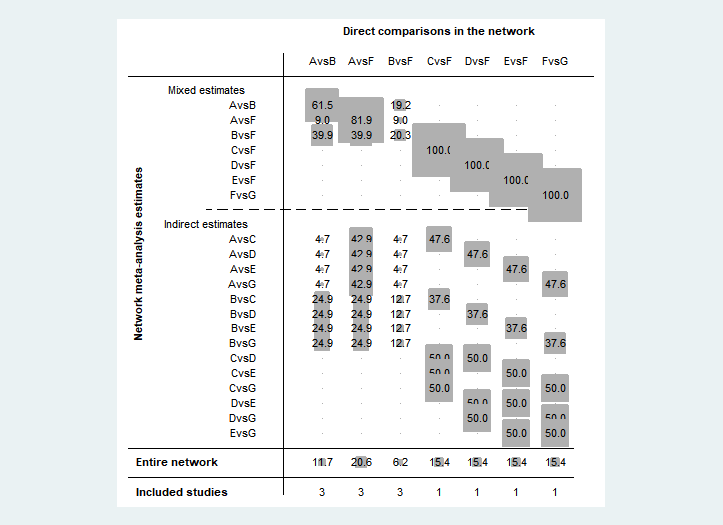

Supplement: Supplementary file 3 [file Image3.tif]

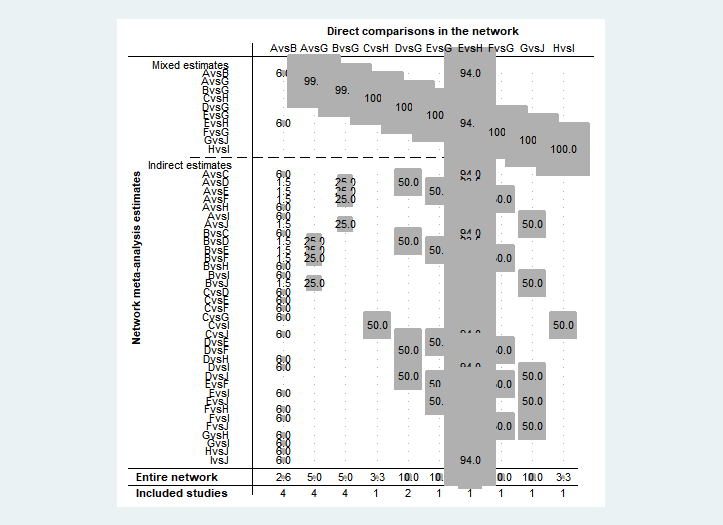

Supplement: Supplementary file 4 [file Image4.tif]

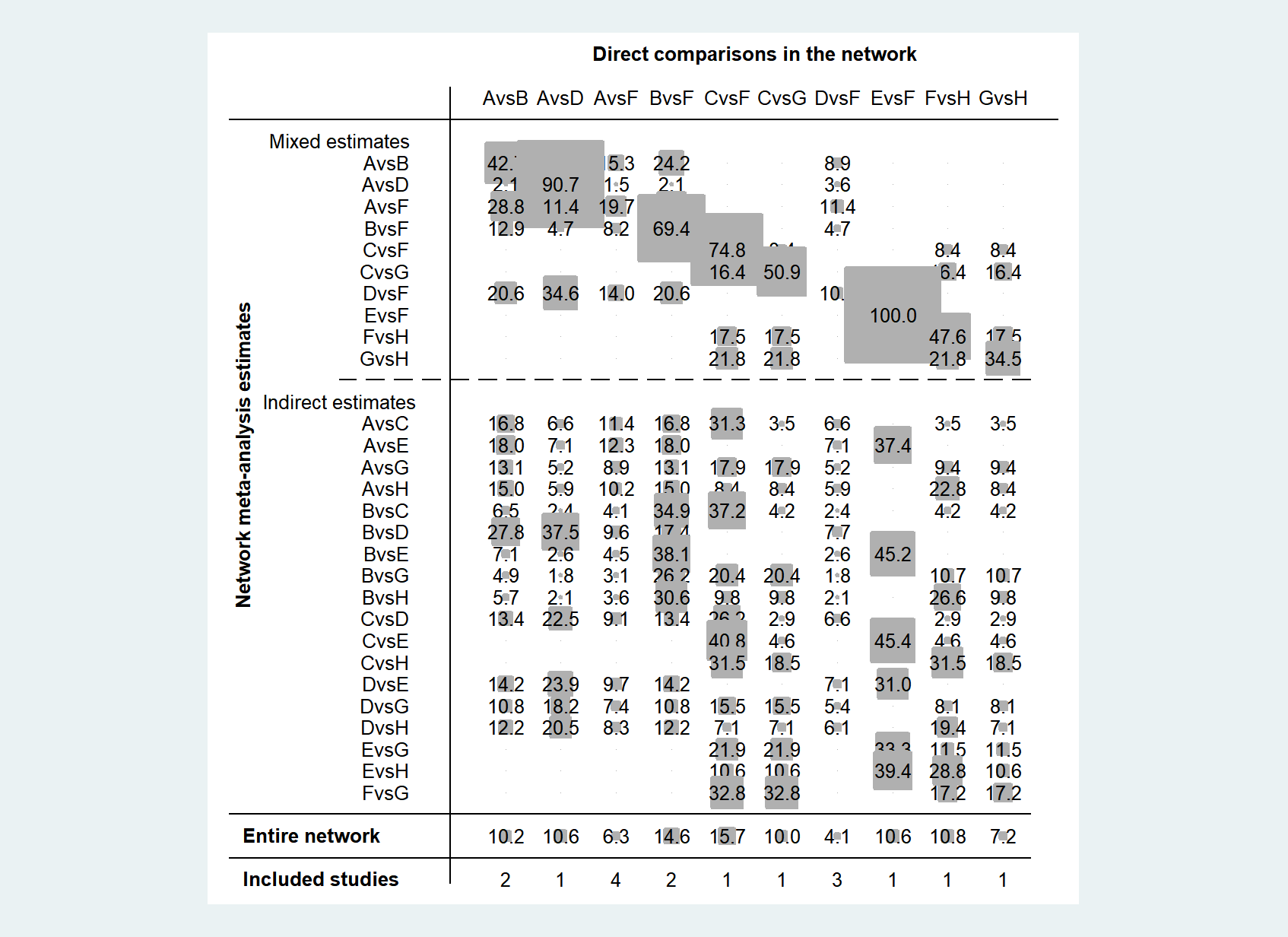

Supplement: Supplementary file 5 [file Image2.tif]

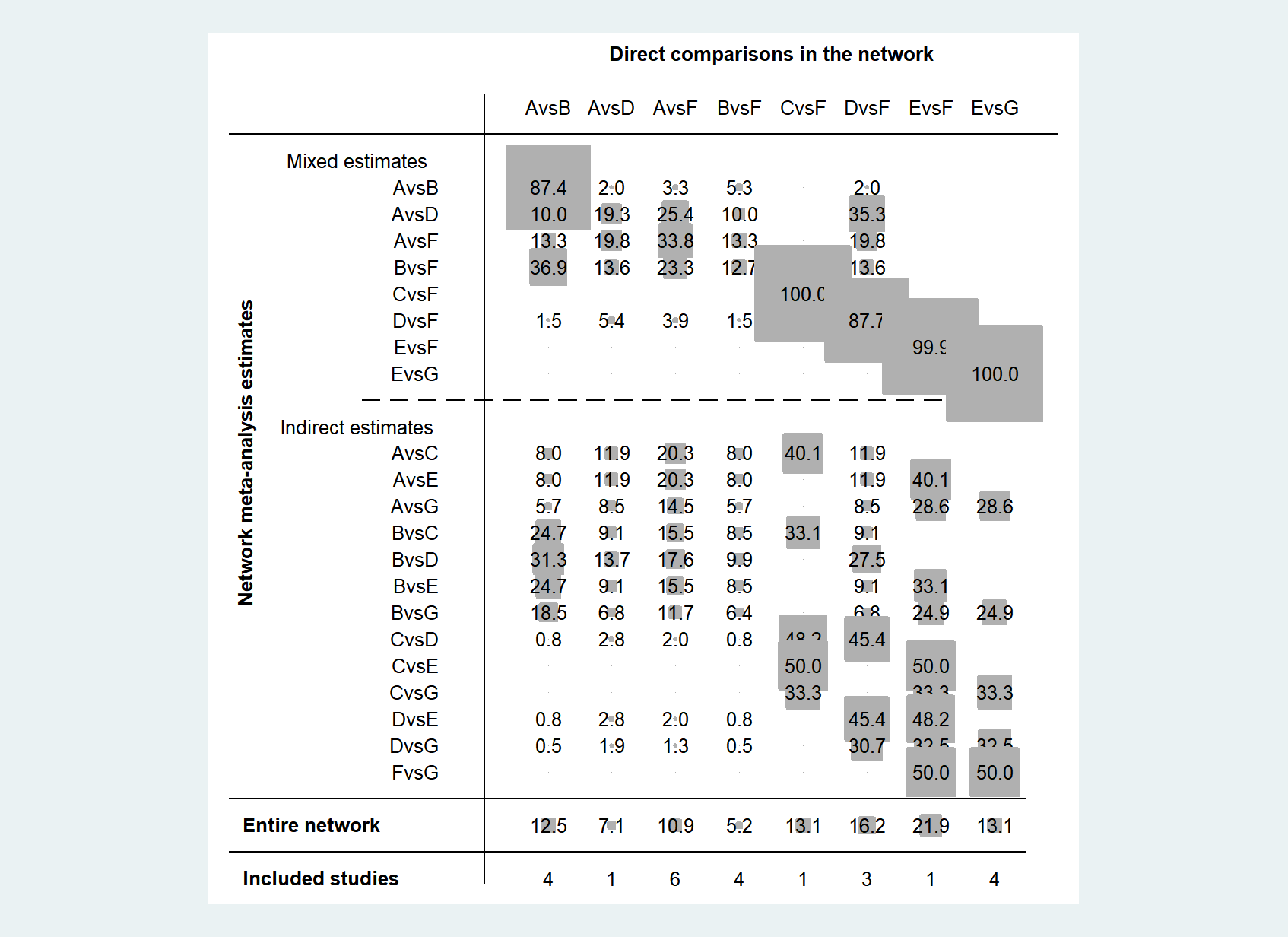

Supplement: Supplementary file 6 [file Image1.tif]

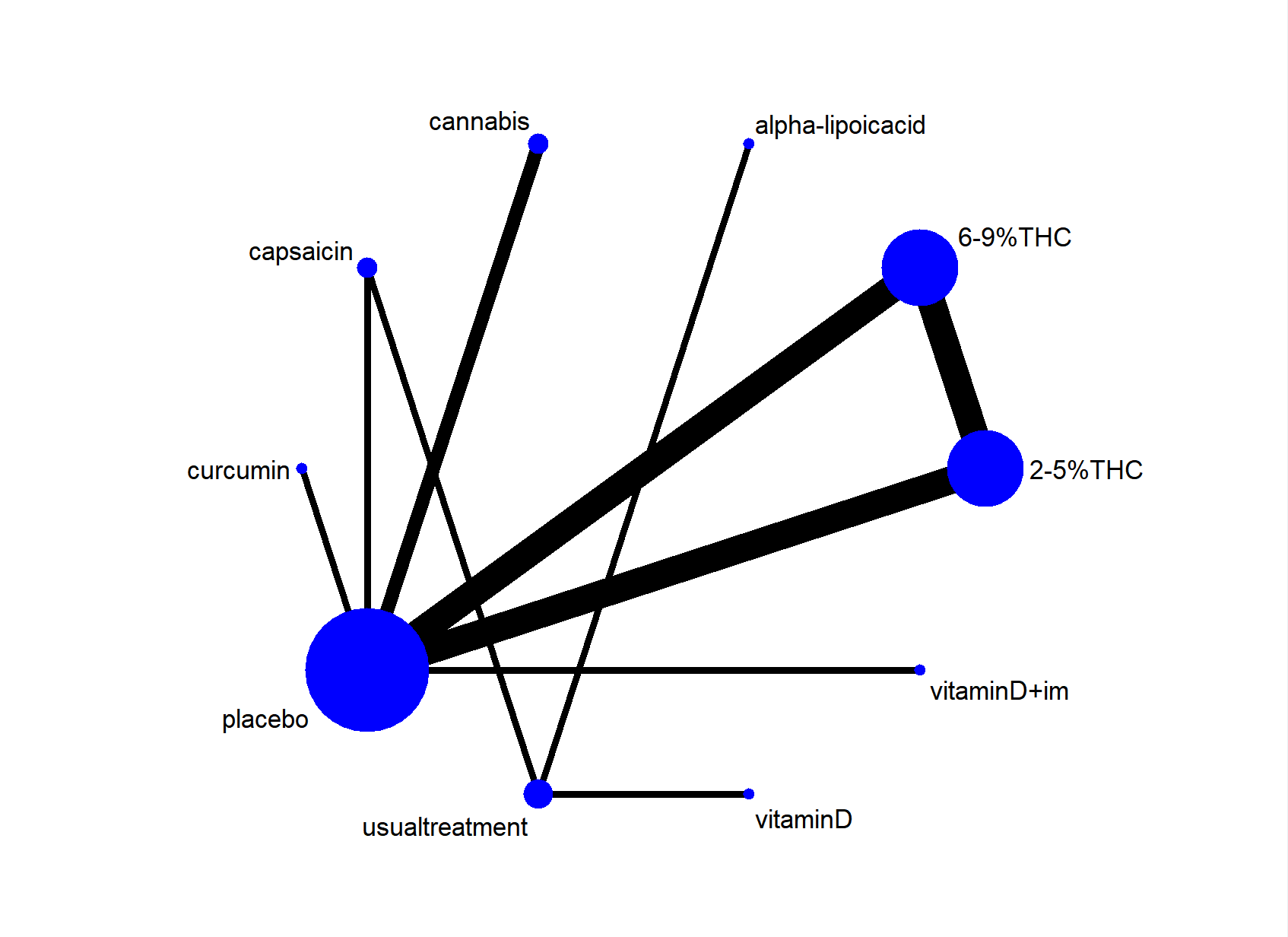

Supplement: Supplementary file 7 [file Image7.tif]

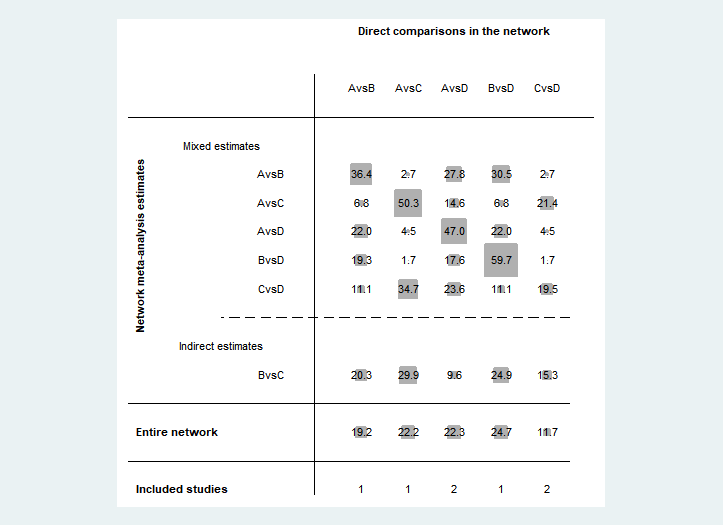

Supplement: Supplementary file 8 [file Image5.tif]
