# Supplementary material for: Systematic review and network meta-analysis of the effects of bioactive compounds on pain intensity and quality of life in neuropathic pain patients
Source: Front Pharmacol. 2025 Oct 17;16:1656400. doi: 10.3389/fphar.2025.1656400 (PMC12576042; doi:10.3389/fphar.2025.1656400)
Supplement: Supplementary file 2 [file Supplementaryfile1.docx]

| Registered Protocol Plan | Final Analysis Action | Deviation Reason |
| --- | --- | --- |
| Intervention: plant extracts | Intervention: bioactive compounds | The intervention terminology was refined from the source-based term "plant extracts" to the mechanism-based term "bioactive compounds" to more accurately encompass the full scope of interventions included in the analysis (e.g., also covering vitamin-related molecules like alpha-lipoic acid and endogenous compounds). This change enhances the pharmacological precision and conceptual clarity of the review. |
| MCIDs for interpreting outcome changes were not predefined | MCIDs for interpreting outcome changes were predefined. | To enhance the clinical interpretation of our findings and align with best practices in meta-analysis, we prospectively defined Minimal Clinically Important Differences (MCIDs) for all primary outcomes prior to data analysis. This addition strengthens the study by allowing for a distinction between statistical significance and clinical relevance, providing a more meaningful context for the estimated treatment effects. |
| Publication bias was assessed via visual inspection of network funnel plots | Publication bias was assessed via visual inspection of network funnel plots and Egger’s test | The assessment method was enhanced by adding Egger's test as a supplementary, quantitative measure to complement the visual inspection of funnel plots. This modification strengthens the objectivity and comprehensiveness of publication bias evaluation by providing statistical evidence alongside graphical assessment. |
| Assessment of heterogeneity | Assessment of heterogeneity and inconsistency | The assessment framework was expanded to include the evaluation of inconsistency, a critical component specific to Network Meta-Analysis. This addition is essential for verifying the validity of the network by statistically testing the agreement between direct and indirect evidence, thereby significantly strengthening the methodological rigor of our analysis. |
| Sensitivity analysis was not involved. | Sensitivity analysis was conducted | In the final analysis, we proactively incorporated a sensitivity analysis, which represents a significant methodological enhancement of the original protocol. This analysis significantly enhanced the reliability of the research conclusions by evaluating the robustness of the results against high-risk biased studies, data conversion methods, and clinical heterogeneity. This improvement makes the analysis more in line with the advanced methodological standards of systematic reviews/meta-analyses |
| GRADE grading was not involved. | GRADE grading was conducted | The GRADE approach was incorporated to enhance the methodological rigor and clinical interpretability of the findings. This addition provides a transparent, systematic framework for rating the certainty of evidence, aligning our study with contemporary standards for high-quality evidence synthesis and supporting more informed clinical decision-making. |

**Table S1. Deviations from the Registered PROSPERO Protocol**

**Table S2. The details of search strategy**

|  | **Search strategy on PubMed** |
| --- | --- |
| **#1** | neuropathic pain[MeSH Major Topic] |
| **#2** | (Neuropathic Pain) OR (neuropathic pains) |
| **#3** | **#1 0R #2** |
| **#4** | ((((((((Capsaicin[MeSH Major Topic]) OR (Curcumin[MeSH Major Topic])) OR (Quercetin[MeSH Major Topic])) OR (Resveratrol[MeSH Major Topic])) OR (Scutellaria baicalensis‌[MeSH Major Topic])) OR (Melatonin[MeSH Major Topic])) OR (Alpha-Lipoic Acid[MeSH Major Topic])) OR (vitamin D[MeSH Major Topic]))OR(Cannabis [MeSH Major Topic])) OR (Ginkgo biloba [MeSH Major Topic]) |
| **#5** | (((((((((((((((((((((((((((((((((((((((‌(E)-Capsaicin) OR (8-Methyl-N-vanillyl-trans-6-nonenamide)) OR (N-Vanillylnonanamide)) OR (Nonylic vanillylamide)) OR (Nonivamide)) OR (Curcumin Phytosome)) OR (Phytosome, Curcumin)) OR (1,6-Heptadiene-3,5-dione, 1,7-bis(4-hydroxy-3-methoxyphenyl)-, (E,E)-)) OR (Diferuloylmethane)) OR (Turmeric Yellow)) OR (Yellow, Turmeric)) OR (Quercetin) OR (Baicalin)) OR (Melatonine)) OR (N-Acetyl-5-Methoxytryptamine)) OR (Anti-Melanin Hormone)) OR (Thioctic Acid)) OR (DL-α-Lipoic Acid‌)) OR (‌5-(1,2-Dithiolan-3-yl)pentanoic Acid)) OR (6,8-Dithiooctanoic Acid)) OR (DL-Thioctic Acid)) OR (Ergocalciferol)) OR (Cholecalciferol‌)) OR (Biflavonoid)) OR (Ginkgo Biloba Biflavone)) ) OR (Resveratrol)) OR (3,4',5-Stilbenetriol)) OR (3,5,4'-Trihydroxystilbene)) OR (3,4',5-Trihydroxystilbene)) OR (trans-Resveratrol)) OR (trans Resveratrol)) OR (Resveratrol-3-sulfate)) OR (Resveratrol 3 sulfate)) OR (SRT 501)) OR (SRT-501)) OR (cis-Resveratrol)) OR (cis Resveratrol)) OR (Resveratrol, (Z)-)) OR (trans-Resveratrol-3-O-sulfate)) OR (trans Resveratrol 3 O sulfate)OR (tetrahydrocannabinol)) OR (cannabidiol) |
| **#6** | **#4 OR #5** |
| **#7** | **#3 AND #6** |

**Table S3. Heterogeneity variance (τ²) and I^2^ statistics for primary outcomes**

| **Outcome** | **Number of study** | **Random-Effects Model (95% CI)** | **Fixed-Effect Model (95% CI)** | **I^2^ (%)** | **τ²** |
| --- | --- | --- | --- | --- | --- |
| VAS | 14 | -1.25 [-1.64, -0.86] | -1.27 [-1.37, -1.17] | 92 | 0.48 |
| NPS | 11 | -1.82 [-2.49, -1.14] | -1.60 [-1.77, -1.43] | 93 | 1.19 |
| HADS | 7 | -1.46 [-2.65, -0.28] | -1.53 [-2.41, -0.65] | 32 | 0.68 |
| PGIC | 11 | -1.13 [ -1.52, -0.75] | -1.33 [-1.50, -1.15 ] | 76 | 0.29 |
| LSEQ | 3 | 0.41 [-1.02, 1.83] | 0.57 [-0.27, 1.41] | 13 | 0.48 |

**Table S4. Consistency test for VAS**

|  | Coef | Std.Err | z | P>\|z\| | [95% Conf. Interval] | |
| --- | --- | --- | --- | --- | --- | --- |
| B VS CON | -.0884373 | .3322692 | -0.27 | 0.790 | -.7396729 | .5627984 |
| C VS CON | -1.554281 | .7221895 | -1.15 | 0.301 | -2.969747 | -.138816 |
| D VS CON | .2235975 | .9459474 | 0.24 | 0.813 | -1.630425 | 2.07762 |
| E VS CON | -1.272262 | 1.043726 | -1.22 | 0.223 | -3.317927 | .7734036 |
| F VS CON | -.7257137 | .7758247 | -0.94 | 0.350 | -2.246302 | .7948748 |
| G VS CON | -.8943131 | 1.101288 | -0.81 | 0.417 | -3.052798 | 1.264172 |

**Table S5. Consistency test for NPS**

|  | Coef | Std.Err | z | P>\|z\| | [95% Conf. Interval] | |
| --- | --- | --- | --- | --- | --- | --- |
| B VS CON | .0828224 | .3519819 | 0.24 | 0.814 | -.6070494 | .7726942 |
| C VS CON | .5637321 | .5793917 | 0.97 | 0.331 | -.5718548 | 1.699319 |
| D VS CON | .6717193 | .9685405 | 0.69 | 0.488 | -1.226585 | 2.570024 |
| E VS CON | .4206155 | .3575418 | 1.18 | 0.239 | -.2801536 | 1.121385 |
| F VS CON | -.0562853 | .5980682 | -0.09 | 0.925 | -1.228477 | 1.115907 |
| G VS CON | -.8943131 | 1.076265 | 0.58 | 0.562 | -1.485527 | 2.733353 |
| H VS CON | .4165186 | .8414116 | 0.50 | 0.621 | -1.232618 | 2.065655 |

**Table S6. Consistency test for HADS**

|  | Coef | Std.Err | z | P>\|z\| | [95% Conf. Interval] | |
| --- | --- | --- | --- | --- | --- | --- |
| B VS CON | -.3894047 | .2184157 | -1.78 | 0.075 | -.8174915 | .0386821 |
| C VS CON | -.4732922 | .4365796 | -1.08 | 0.278 | -1.328972 | .3823881 |
| D VS CON | .9665877 | .4560006 | 1.12 | 0.134 | .0728429 | 1.860333 |
| E VS CON | 1.026455 | .4370389 | 0.35 | 0.619 | .1698744 | 1.883035 |
| F VS CON | .227706 | .2157973 | 0.94 | 0.311 | -.804751 | 1.650661 |
| G VS CON | .492295 | .3322881 | 1.15 | 0.201 | -.6450256 | 1.339564 |

**Table S7. Consistency test for PGIC**

|  | Coef | Std.Err | z | P>\|z\| | [95% Conf. Interval] | |
| --- | --- | --- | --- | --- | --- | --- |
| B VS CON | 1.10366 | 1.111043 | 0.99 | 0.321 | -1.073945 | 3.281266 |
| C VS CON | 1.05527 | 3.933279 | 0.27 | 0.788 | -6.653815 | 8.764355 |
| D VS CON | -1.5328 | 1.917388 | -0.80 | 0.424 | -5.290813 | 2.225212 |
| E VS CON | -.3249034 | 2.481905 | -0.13 | 0.896 | -5.189348 | 4.539541 |
| F VS CON | -.648019 | 2.481353 | -0.26 | 0.794 | -5.511382 | 4.215344 |
| G VS CON | -2.930669 | 1.12572 | -1.60 | 0.097 | -5.137039 | 2.7242987 |
| H VS CON | -.6538024 | 3.278858 | -0.20 | 0.842 | -7.080246 | 5.772641 |
| I VS CON | -.5177699 | 2.482883 | -0.21 | 0.835 | -5.38413 | 4.348591 |

**Table S8. Consistency test for LSEQ**

|  | Coef | Std.Err | z | P>\|z\| | [95% Conf. Interval] | |
| --- | --- | --- | --- | --- | --- | --- |
| B VS CON | .7901879 | .602322 | 1.31 | 0.190 | -.3903415 | 1.970717 |
| C VS CON | -.4782148 | .5244277 | -0.91 | 0.362 | -1.506074 | .5496446 |
| D VS CON | .2005362 | .4399206 | 0.46 | 0.649 | -.6616922 | 1.062765 |

**Table S9. Node-Splitting Analysis for VAS.**

**Table S10. Node-Splitting Analysis for NPS.**

**Table S11. Node-Splitting Analysis for HADS.**

**Table S12. Node-Splitting Analysis for PGIC.**

**Table S13. Node-Splitting Analysis for LSEQ.**

**Table S14. NPS SUCRA Ranking**

| Treatment | SUCRA | PrBest | MeanRank |
| --- | --- | --- | --- |
| placebo | 16.9 | 0.5 | 7.6 |
| 2-5%THC | 85.6 | 48.7 | 2.2 |
| cannabis | 48.1 | 0.1 | 5.1 |
| 6-9%THC | 74.3 | 9.5 | 3.1 |
| usual+treatment | 23.5 | 1.1 | 7.1 |
| alpha+lipoicacid | 64.1 | 7.2 | 3.9 |
| vitaminD | 53.8 | 14.5 | 4.7 |
| vitaminD+im | 51.2 | 6.9 | 4.9 |
| curcumin | 32.4 | 11.4 | 6.4 |

**Table S15. PGIC SUCRA Ranking**

| Treatment | SUCRA | PrBest | MeanRank |
| --- | --- | --- | --- |
| placebo | 24.7 | 0.0 | 7.8 |
| 2-5%THC | 86.3 | 37.4 | 2.2 |
| 6-9%THC | 64.8 | 11.8 | 4.2 |
| cannabis | 54.1 | 8.6 | 5.1 |
| capsaicin | 51.2 | 9.8 | 5.4 |
| usual+treatment | 47.0 | 3.2 | 5.8 |
| alpha+lipoicacid | 32.7 | 6.0 | 7.1 |
| vitaminD | 42.5 | 0.4 | 6.2 |
| vitaminD+im | 46.2 | 13.3 | 5.8 |
| curcumin | 50.4 | 9.5 | 5.5 |

**Table S16. Egger's Test results for primary outcomes.**

| Study Set | Coefficient | Std. Err | t-value | p-value | [95% Conf. Interval] |
| --- | --- | --- | --- | --- | --- |
| **VAS** |  |  |  |  |  |
| bias | 0.12 | 0.08 | 1.50 | 0.138 | [-0.04, 0.28] |
| _cons | -0.25 | 0.15 | -1.67 | 0.098 | [-0.55, 0.05] |
| **NPS** |  |  |  |  |  |
| bias | -0.08 | 0.07 | -1.14 | 0.256 | [-0.22, 0.06] |
| _cons | 0.18 | 0.12 | 1.50 | 0.136 | [-0.06, 0.42] |
| **HADS** |  |  |  |  |  |
| bias | 0.05 | 0.06 | 0.83 | 0.408 | [-0.07, 0.17] |
| _cons | -0.12 | 0.10 | -1.20 | 0.232 | [-0.32, 0.08] |
| **PGIC** |  |  |  |  |  |
| bias | -0.15 | 0.09 | -1.67 | 0.097 | [-0.33, 0.03] |
| _cons | 0.22 | 0.16 | 1.38 | 0.169 | [-0.10, 0.54] |
| **LSEQ** |  |  |  |  |  |
| bias | 0.09 | 0.07 | 1.29 | 0.199 | [-0.05, 0.23] |
| _cons | -0.15 | 0.13 | -1.15 | 0.250 | [-0.41, 0.11] |
